# ESSENTIAL MEIOTIC ENDONUCLEASE 1 is required for chloroplast development and DNA repair in rice

**DOI:** 10.1111/pbi.70101

**Published:** 2025-05-07

**Authors:** Yanxin Du, Yang Li, Weijiang Tang, Weiping Mo, Tingting Ma, Rongcheng Lin

**Affiliations:** ^1^ Key Laboratory of Photobiology, Institute of Botany Chinese Academy of Sciences Beijing China; ^2^ Biotechnology Institute Xianghu Laboratory Hangzhou China; ^3^ Present address: State Key Laboratory of Plant Environmental Resilience China Agricultural University Beijing China; ^4^ Present address: National Crop Genebank, Institute of Crop Science Chinese Academy of Agricultural Sciences Beijing China; ^5^ Present address: Institute of Genetics and Developmental Biology Chinese Academy of Sciences Beijing China

**Keywords:** chloroplast development, DNA repair, rice

## Abstract

Chloroplast development is fundamental to photosynthesis and plant growth but is sensitive to environmental stress. Chloroplast development and division require genome stability and DNA repair, but the underlying mechanisms have been unclear. Using a forward genetic approach, we identified the striped‐leaf mutant *k48* in the rice (*Oryza sativa* L. *japonica*) cultivar KY131 background. *k48* displayed defects in chloroplast development and photosynthesis, especially under high‐light conditions. Genetic and complementation studies revealed that the loss of ESSENTIAL MEIOTIC ENDONUCLEASE 1 (EME1) is responsible for the defects in *k48*. Transcriptomic analysis showed that OsEME1 globally regulates the expression of genes involved in photosynthesis and DNA repair. Furthermore, mutations in *OsEME1* led to cell cycle arrest and a DNA damage response. An *in vitro* endonuclease activity assay indicated that OsEME1 directly binds to and cleaves DNA substrates with a specific structure and that four conserved amino acids are required for its activity. Notably, OsEME1 targeted DNA fragments of rice *GOLDEN‐LIKE 1* (*GLK1*) and *GLK2*. We also demonstrated that OsEME1 interacts with the structure‐specific endonuclease methyl methanesulfonate (MMS) and UV‐SENSITIVE PROTEIN 81 (MUS81). This study highlights the role of OsEME1 in regulating chloroplast development by modulating homologous recombination repair in response to damage to double‐stranded DNA.

## Introduction

Chloroplasts are plant organelles that originated from an ancient endosymbiosis. Chloroplasts are indispensable for plant and algal growth, not only for performing photosynthesis but also for carrying out biochemical processes such as the biosynthesis of pigments, amino acids and fatty acids and sensing environmental stimuli (Jarvis and López‐Juez, 2013). Chloroplast biogenesis and development are regulated by internal developmental cues and environmental signals, which vary according to tissue type and the life stage of the plant (Pogson *et al*., 2015; Pogson and Albrecht, 2011). Endogenous plant hormones such as brassinosteroids, cytokinins, auxins, gibberellins and strigolactones promote chloroplast development, particularly during early stages of plant development (Cackett *et al*., 2022; Müller and Munné‐Bosch, 2021).

Light is crucial for chloroplast development in angiosperms because it initiates the expression of hundreds of chloroplast‐related genes as well as chlorophyll biosynthesis (Huq *et al*., 2004; Shin *et al*., 2009). Transcription factors belonging to several families, including GOLDEN2 (G2) LIKE 1 and 2 (GLK1 and GLK2), GATA NITRATE‐INDUCIBLE CARBON‐METABOLISM‐INVOLVED (GNC), CYTOKININ‐RESPONSIVE GATA FACTOR 1 (CGA1) and LLM‐domain B‐GATAs, act downstream of light in many plants to regulate the size and abundance of chloroplasts (Behringer and Schwechheimer, 2015; Fitter *et al*., 2002; Hall *et al*., 1998; Hua *et al*., 2021; Langdale and Kidner, 1994; Wang *et al*., 2013, 2017; Waters *et al*., 2008). GLKs regulate the expression of genes related to chloroplast development and chlorophyll biosynthesis in the light and dark directly by binding to target genes or indirectly by responding to chloroplast retrograde signals (Tu *et al*., 2022). Light is also responsible for chloroplast protein degradation. Under high‐light conditions, photoinhibition is activated and reactive oxygen species (ROS) are produced and accumulate in chloroplasts, resulting in an imbalanced energy distribution between photosystem I (PSI) and photosystem II (PSII) and a rapid decline in photosynthetic efficiency (Asada, 2006; Walters, 2005; Wang *et al*., 2024).

Plants are widely exposed to environmental stress conditions owing to their sessile nature. Genomic DNA is at constant risk of damage from endogenous substances, environmental radiation and chemical stressors. DNA damage caused by different endogenous and environmental agents can be categorized into two major classes: single‐strand breaks (SSBs) and double‐strand breaks (DSBs) (Friedberg *et al*., 2004; Schubert, 2021). DNA replication errors, spontaneous base deamination and alkylating agents induce the formation of SSBs, which occur frequently in cells and are easily repaired through mismatch repair or base‐excision repair (Caldecott, 2022; Chatterjee and Walker, 2017).

Double‐strand breaks are induced by UV‐B or UV‐C radiation, ionizing radiation, crosslinking agents or oxidative damage. Although they do not occur as frequently as SSBs, DSBs disrupt genomic integrity and induce cell cycle arrest, cellular degradation and even cell death (Duan *et al*., 2020; Jackson and Bartek, 2009; Schubert, 2021; Woodson, 2019). In mammalian cells, DSBs can be repaired via several mechanisms: canonical non‐homologous end‐joining (c‐NHEJ), alternative non‐homologous end‐joining (alt‐NHEJ), homologous recombination (HR) and single‐strand annealing (Densham and Morris, 2019; Kowalczykowski, 2015; Pannunzio *et al*., 2018).

Plants also possess DNA damage response pathways that transport signals and defend against damage. Cell cycle arrest and DNA repair or cell autophagy are activated by a series of kinase cascades involving ATAXIA‐TELANGIECTASIA‐MUTATED (ATM), ATAXIA TELANGIECTASIA AND RAD3‐RELATED (ATR) and phosphoinositide 3‐kinase‐like kinases (PIKKs) (Hu *et al*., 2016; Zhang *et al*., 2015). Chloroplast division generally occurs during the S phase of the cell cycle and is promoted by cytokinins, light and cell expansion. Several cell‐cycle‐related proteins (such as CDT1a) are recruited to coordinate the cell cycle and plastid division in Arabidopsis (*Arabidopsis thaliana*) (Domenichini *et al*., 2012; Pedroza‐Garcia *et al*., 2016; Raynaud *et al*., 2005). The cell cycle also regulates chloroplast differentiation and chloroplast genomic stability after plastid division. The WHIRLY (WHY) proteins WHY1 and WHY3 localize to chloroplasts, where they maintain chloroplast genome stability by stabilizing single‐stranded DNA and guiding it through conservative repair mechanisms (Cappadocia *et al*., 2010; Maréchal *et al*., 2009). Chloroplast‐localized CND1 is involved in maintaining the stability of the chloroplast genome by interacting with WHY1. CND1 interacts with MCM4 in the nucleus during the regulation of the cell cycle (Jin *et al*., 2023). RECA proteins are key factors mediating the HR repair pathway of DNA in chloroplasts (Shedge *et al*., 2007). RECA1/WHY1/WHY3 mediate the cell cycle through SOG1/SMR5/SMR7 and thus regulate chloroplast genome stability (Duan *et al*., 2020; Lepage *et al*., 2013; Zampini *et al*., 2015). However, how chloroplast division and differentiation and the cell cycle are regulated in rice (*Oryza sativa*) is largely unknown.

Holliday junctions (HJs) accumulate rapidly in plants as recombination intermediates during the repair of damaged double‐stranded DNA, when transcription is interrupted by strand exchange between a damaged chromosome and its sister chromatid. Eme1 [methyl methanesulfonate 4 (Mms4) in *Saccharomyces cerevisiae*] forms a heterodimeric endonuclease with Mus81 in *Schizosaccharomyces pombe*, cleaving branched DNA substrates such as HJs, 3′‐DNA flaps and replication forks (Boddy *et al*., 2001; Haber and Heyer, 2001). Human EME1 and EME2 also form a complex with MUS81 and cleave HJs, 3′‐DNA flaps and replication forks (Ciccia *et al*., 2003; Oğrünç and Sancar, 2003; Pepe and West, 2014). Eme1 (EME1) lacks endonuclease activity; its interaction with Mus81 (MUS81) regulates the endonucleolytic activity of Mus81 in yeast and human cells. Mus81–Mms4 is activated by cell division cycle 5 (Cdc5)‐, cyclin‐dependent kinase 1 (Cdk1)‐ and Dumb‐bell‐forming 4 (Dbf4)‐dependent phosphorylation of Mms4 during the G2/M transition in *S. cerevisiae* (Gallo‐Fernández *et al*., 2012; Princz *et al*., 2017; Szakal and Branzei, 2013).

The accumulation of hyper‐activated Mus81–Mms4 is restricted to mitosis through the SUMOylation and ubiquitination of specifically phosphorylated Mms4 for degradation by the proteasome (Waizenegger *et al*., 2020). In *S. pombe*, the upregulation of Mus81–Eme1 depends on the phosphorylation of Eme1 by cell division cycle 2 (Cdc2) and Eme1 is further phosphorylated by DNA damage checkpoint factors such as Rad3 in response to DNA damage (Dehé *et al*., 2013; Giaccherini *et al*., 2022). Synthetic lethal of unknown function 4 (SLX4) provides a scaffold for multiple DNA repair factors in human cells. The C terminus of SLX4 binds to catalytic SLX1 to form the SLX4–SLX1 complex, which interacts with MUS81–EME1 via a direct interaction between MUS81 and SLX4 (Garner *et al*., 2013; Nowotny and Gaur, 2016; Young and West, 2021). SLX4 is phosphorylated by Cyclin‐dependent kinase 1 (CDK1) during the G2/M transition and upregulates the activity of the MUS81–EME1 complex (Duda *et al*., 2016; Payliss *et al*., 2022). The homologous proteins AtEME1A and AtEME1B are recruited to Holliday junction substrates in Arabidopsis; these proteins bind to and cleave their substrates at nicks in the DNA strands (Geuting *et al*., 2009). However, the mechanism that underlies HR repair in rice remains largely unknown.

Here, we cloned the rice *EME1* (*OsEME1*) gene via genetic screening of the *k48* mutant, which showed defects in chloroplast development and was hypersensitive to DNA‐damaging agents. We determined that OsEME1 regulates the expression of genes involved in chloroplast development and DNA repair genome‐wide. OsEME1 directly binds to and cleaves Y12, pre‐X12 and X12, which are typical substrates after HR repair of DNA damage (Boddy *et al*., 2001; Parsons *et al*., 1990). This study reveals essential roles for OsEME1 in regulating chloroplast development and DNA repair in rice.

## Results

### The *k48* rice mutant has defects in chloroplast development

**Figure 1:**
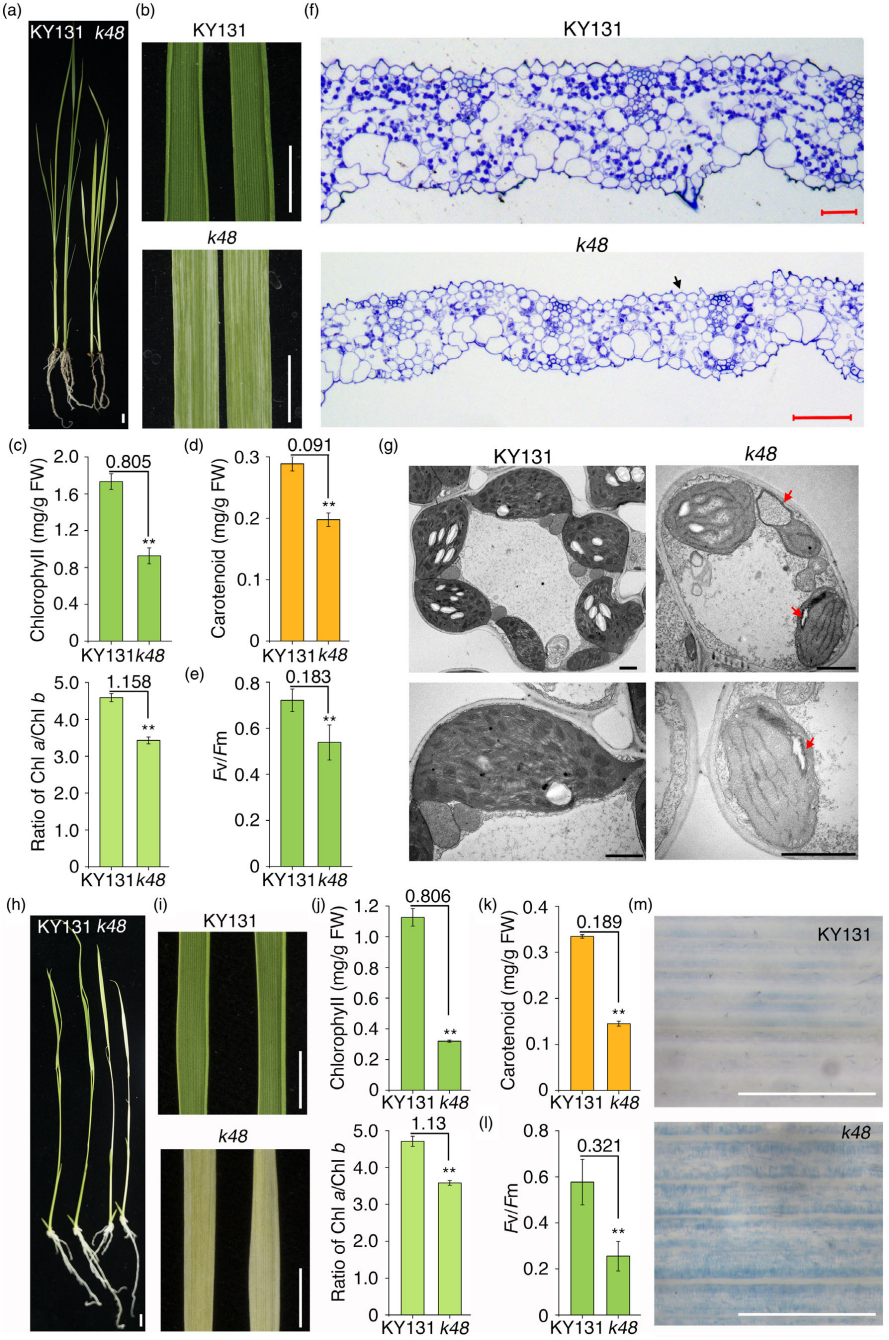
The *k48* mutant shows severe defects in chloroplast development. (a) Seedling phenotype of the *k48* mutant and Kongyu 131 wild type (KY131) grown under normal light conditions (~300 μmol m^−2^ s^−1^) at 28 °C for 12 days. Bar, 1 cm. (b) Leaf phenotype of the seedlings shown in (a). Bars, 0.5 cm. (c) Chlorophyll levels of *k48* and KY131. FW, fresh weight. (d) Carotenoid contents of *k48* and KY131. (e) Maximum efficiency of PSII photochemistry (*F*v/*F*m) of *k48* and KY131. (f) Semithin section of leaves. Black arrow indicates blade section without chloroplasts. Bar, 50 μm. (g) Transmission electron microscopy of chloroplasts. Red arrows indicate chloroplast autophagy. Bars, 1 μm. For (c–g), plants were grown under the same conditions shown in (a). (h) Phenotype of seedlings grown under normal light conditions at 28 °C for 9 days followed by high‐light treatment (~700 μmol m^−2^ s^−1^) at 28 °C for 3 days. Bar, 1 cm. (i) Leaf phenotype of the seedlings shown in (h). Bars, 0.5 cm. (j) Chlorophyll contents of *k48* and KY131. (k) Carotenoid contents of *k48* and KY131. (l) *F*v/*F*m of *k48* and KY131. (m) Trypan blue staining of *k48* and KY131. For (j–m), plants were grown under the same conditions as in (h). For (c–e) and (j–l), values on the bars indicate difference value between WT and mutant, which were obtained by subtracting the mutant data from the wild‐type data. Data are mean ± SD from three biological replicates. Asterisks indicate significant differences using Student's *t*‐test (**P < 0.01).

To identify factors that regulate chloroplast development or chlorophyll biosynthesis in rice, we generated a mutant pool in the *japonica* rice cultivar Kongyu 131 (KY131) background using ethyl methyl sulfone (EMS). Among the approximately 5000 M1 lines generated, nearly 50 mutants showed defects in leaf colour, and their phenotypes were inherited by their M2 progeny. One of these mutants, *k48*, displayed striped albino leaves and reduced growth when grown under long‐day conditions (16‐h light/8‐h dark) with normal light intensity (NL; ~300 μmol/m^2^/s) (Figure 1a,b). Compared with the wild type (KY131), *k48* accumulated significantly lower levels of chlorophylls and carotenoids (Figure 1c,d). The maximum efficiency of PSII photochemistry (*F*v/*F*m) was much lower in *k48* (0.53) than in KY131 (0.72) (Figure 1e). Analysis of semithin sections of leaves revealed thinner leaves with many fewer chloroplasts and a looser cell arrangement in *k48* than in KY131 (Figure 1f). Leaf cells of *k48* exhibited severe defects in chloroplast development, with a lack of grana organization and lamellar structures and chloroplast autophagy in most mesophyll cells. By contrast, KY131 cells displayed typical thylakoid structures and chloroplast numbers (Figure 1g and Figure S1a). These observations indicate that chloroplast development is impaired in the *k48* mutant.

### High‐light stress enhances the phenotype of *k48*

High light often causes photo‐oxidative stress to plants and chloroplasts (Sandalio and Romero‐Puertas, 2015). When plants were grown under long‐day cycles of NL for 9 days and then exposed to relatively high light intensity (HL; ~700 μmol m^−2^ s^−1^) under the same long‐day cycles for 3 days, the *k48* plants exhibited strong phenotypes, with albino leaves and strongly reduced chlorophyll and carotenoid contents compared to KY131 (Figure 1h–k). The *F*v/*F*m of *k48* (0.26) was less than half that of KY131 (0.58) under HL conditions (Figure 1l). Consistently, trypan blue staining showed that *k48* exhibited severe cell death compared to KY131 under HL conditions (Figure 1m).

To examine the molecular defects of *k48*, we performed reverse transcription quantitative PCR (RT‐qPCR) to compare the expression levels of chloroplast‐encoded genes between *k48* and KY131 grown under NL and HL conditions. The expression levels of these photosynthesis‐regulated genes (encoding PSI or PSII reaction centre protein subunits) were significantly lower in *k48* than in KY131 in seedlings grown under both light conditions. However, the expression levels of rRNA‐related genes, such as *rpl12* and *rpl14*, were higher in *k48* than in KY131 under NL conditions (Figure S1b). Strikingly, the transcript levels of all rRNA‐related genes except *ndhF* examined were higher in *k48* than in KY131 under HL conditions (Figure S1c). Conversely, BN‐Gel analysis showed that the PSI and PSII complexes were severely damaged in *k48* under both light conditions (Figure S1d) and levels of the subunits of photosynthetic protein complexes were significantly reduced in *k48* compared to KY131, especially under HL (Figure S1e). These data indicate that high‐light stress enhances the phenotypes of *k48*.

### Cloning of Os*EME1*

To determine whether *k48* was a recessive or dominant mutant, we backcrossed *k48* with KY131. The F2 progenies included plants with green leaves versus pale leaves that segregated at an approximately 3:1 ratio (208:72), indicating that the *k48* phenotype originated from a single recessive mutation in a nuclear gene. We assigned 48 progenies with striped albino leaves and 48 with green leaves to the Pale1‐pool and WT‐pool, respectively, and subjected them to DNA resequencing. The mean coverage depth for the parents and the two pools was 20×. A comparison of the sequences to the ‘Nipponbare’ reference genome resulted in the identification of 321 840 single‐nucleotide polymorphisms (SNPs); this number was reduced to 4394 by trimming and filtering.

**Figure 2:**
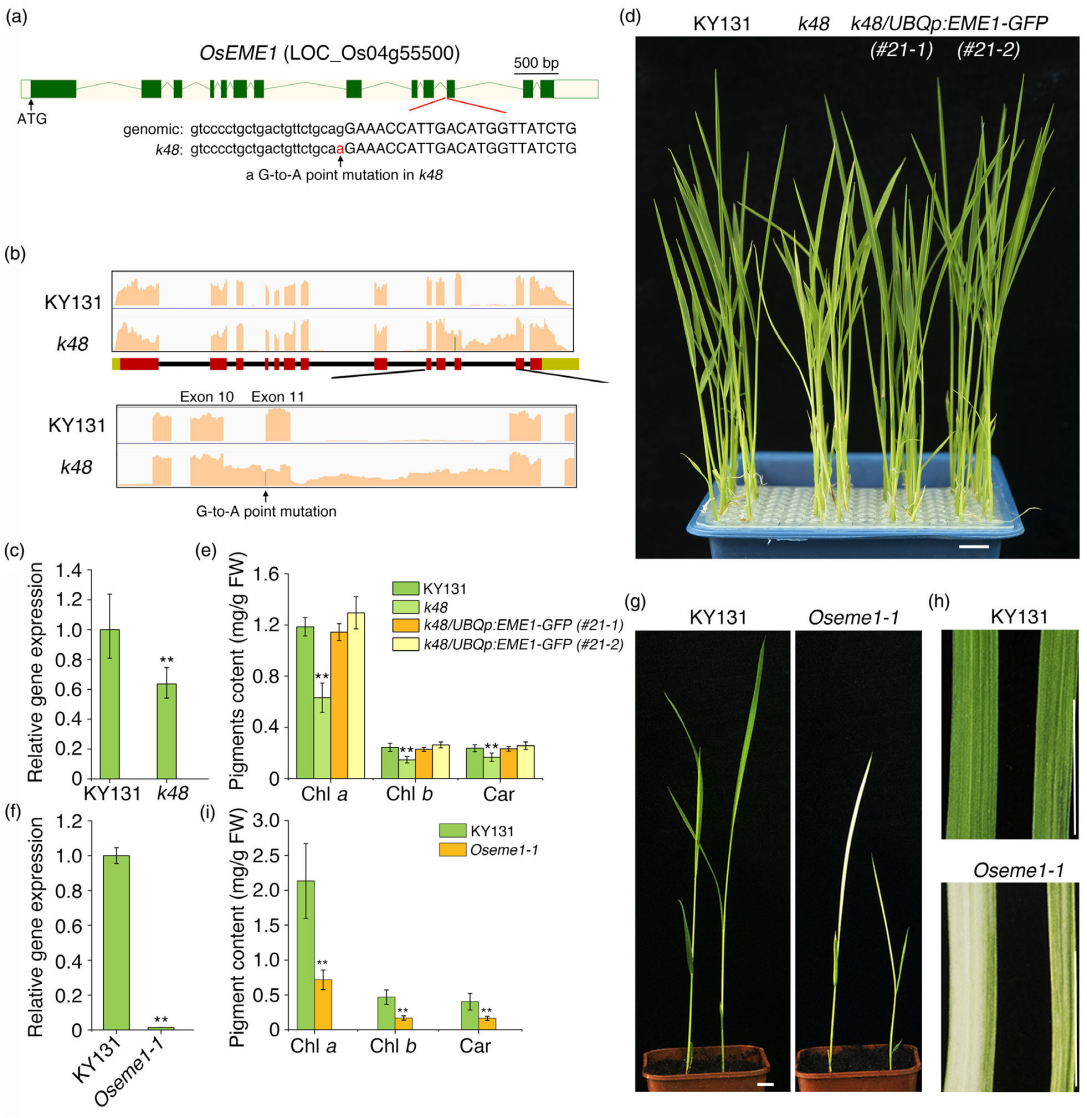
Characterization of the mutants and transgenic complementation lines of *OsEME1*. (a) Diagram of *OsEME1* gene structure in wild‐type KY131 and position of the point mutation in *k48*. Green bars indicate exons, while lines denote introns. A point mutation (in red) at the end of the 10th intron was identified in the *k48* mutant. (b) The abundance of 10th intron and 11th intron retention was significantly increased in the *k48* mutant compared to wild‐type KY131. (c) *OsEME1* expression in *k48* and KY131. (d) Phenotype of two independent complementation lines of *k48/UBQp:OsEME1‐GFP* grown under normal light conditions as shown in Figure 1a. Bar, 1 cm. (e) Pigment contents of the seedlings shown in d. (f) *OsEME1* transcript levels in the *Oseme1* mutant and KY131. (g, h) Seedling (g) and leaf (h) morphology of the *Oseme1* mutant and KY131 grown under normal light conditions. Bars, 1 cm. (i) Pigment contents of the seedlings shown in g. For (c, e, f and i), data are mean ± SD from three biological replicates. Asterisks indicate significant differences using Student's *t*‐test (**P < 0.01).

We selected these 4394 high‐quality SNPs, which were homozygous in each parent and polymorphic between the parents, and subjected them to BSA‐seq analysis using *q*‐values (corrected Fisher's exact test *P*‐values). Three significant peaks in the SNP‐Index distribution spanned intervals of 8.98 Mb (26.27–35.25 Mb), 8.01 Mb (31.43–39.44 Mb) and 8.41 Mb (25.24–33.65 Mb) on chromosome 2, 4 and 7, respectively (Figure S2a). Among the 148 candidate SNPs identified in these three regions, 10 candidate SNPs were chosen after filtering using *q*‐values (Figure S2b). Map‐based cloning together with PCR genotyping revealed the presence of a G‐to‐A mutation at the junction site between the tenth intron and the eleventh exon of LOC_Os04g55500 in *k48* (Figure 2a). LOC_Os04g55500 encodes an endonuclease with homology to EME1 in Arabidopsis (Geuting *et al*., 2009). The G‐to‐A mutation caused mis‐splicing and intron retention in *OsEME1*, resulting in the premature translation termination of *OsEME1* in *k48* (Figure 2b and Figure S3). The total Os*EME1* transcript levels were significantly reduced in *k48*, as revealed by RT‐qPCR (Figure 2c).

To confirm that the phenotype of *k48* was caused by the mutation in *OsEME1*, we introduced the wild‐type *OsEME1* sequence fused with *GFP* (encoding green fluorescent protein) driven by the ubiquitin promoter (*UBQp::OsEME1‐GFP*) into *k48*. In immunoblot analysis, the transgenic plants produced one band containing the GFP fusion protein (Figure S4a). The exogenous expression of *OsEME1‐GFP* rescued the pale leaf phenotype and low chlorophyll and carotenoid contents of *k48* (Figure 2d,e). Moreover, we generated an *Oseme1* mutant using CRISPR/Cas9‐mediated gene editing. The mutant, which contains a 467‐nt deletion from bp 1764 to 2230 and a G‐to‐T transversion at bp 2233, was designated as *Oseme1‐1* (Figure S4b). *OsEME1* transcript was undetectable in *Oseme1‐1* (Figure 2f). *Oseme1‐1* seedlings exhibited severe phenotypes, with striped albino leaves (Figure 2g,h). The chlorophyll and carotenoid contents were significantly reduced in *Oseme1‐1* compared to KY131 (Figure 2i). These data confirm the notion that the mutation in *OsEME1* leads to defects in chloroplast development and that the *k48* phenotype is indeed caused by the loss of *OsEME1*.

### Characterization of *OsEME1*

Sequence analysis revealed that OsEME1 possesses three disordered regions (aa 1–94, 107–168 and 220–326), a coiled‐coil domain (aa 291–355) and an ERCC4 domain (aa 391–592) (Figure S5a). Phylogenetic analysis indicated that the endonuclease EME1 is conserved in mammals, yeasts and plants including Arabidopsis, rice and maize (*Zea mays*) (Figure S6), suggesting that EME1 might play universal roles in all kingdoms of life. Os*EME1* was expressed ubiquitously in young shoots, leaves and roots (Figure S5b). In a transient expression assay in Arabidopsis protoplasts, the OsEME1‐GFP fusion protein localized to the nucleus and overlapped closely with the nuclear protein H2B‐mCherry (Figure S5c), which is consistent with its putative biochemical role as an endonuclease.

### OsEME1 globally regulates the expression of genes related to chloroplast development, photosynthesis and DNA damage repair

**Figure 3:**
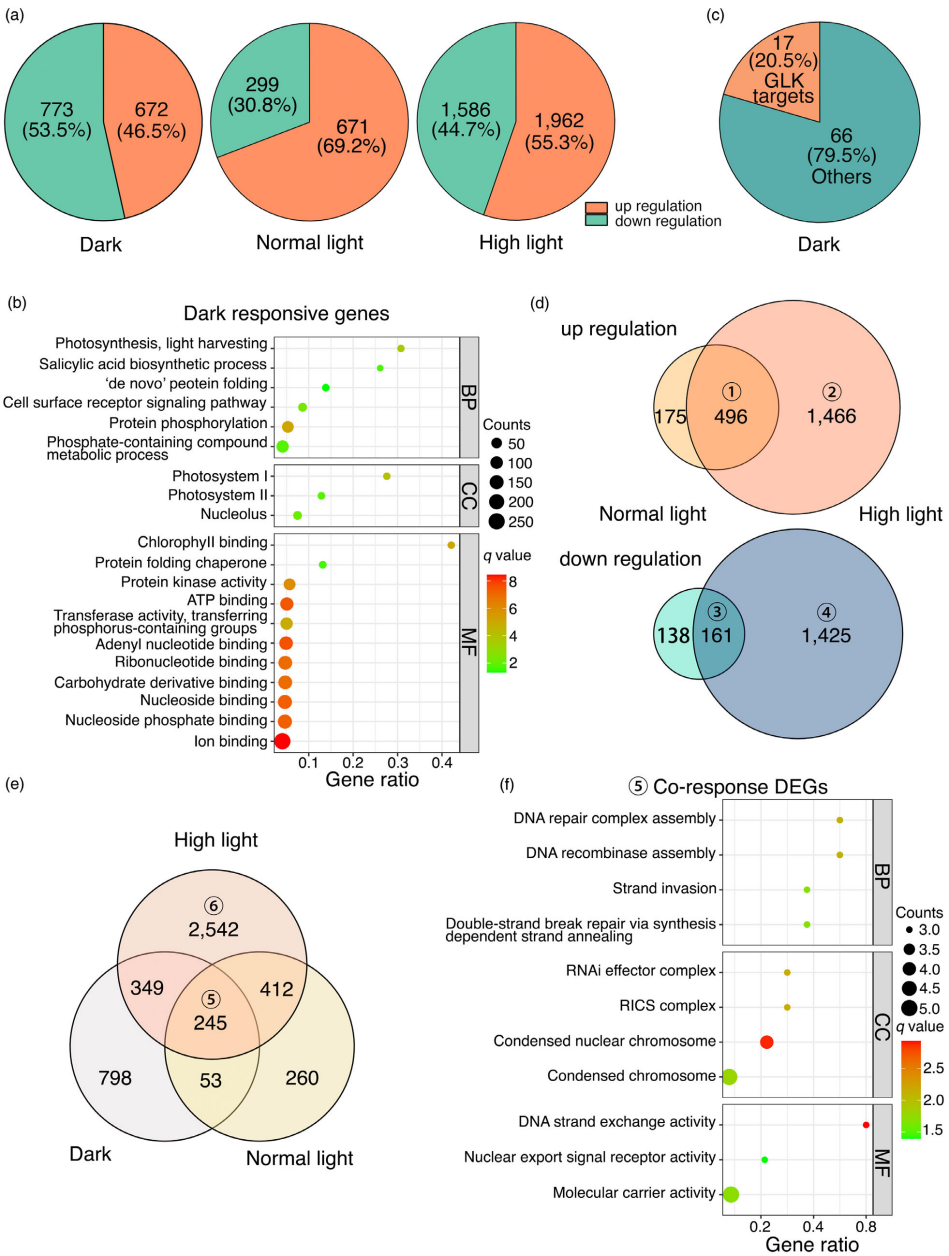
Transcriptomic analysis of the *k48* mutant. (a) Pie charts showing the number and percentage of up‐ and downregulated genes in *k48* compared to KY131. KY131 and *k48* seedlings were grown under a long‐day photoperiod (16‐h light/8‐h dark) for 6 days and one batch of samples was collected at the end of the dark period (designated as dark). The other two batches were exposed to NL (~300 μmol m^−2^ s^−1^) or HL (~700 μmol m^−2^ s^−1^) conditions for 5 h before harvesting. (b) Gene Ontology (GO) analysis of differentially expressed genes (DEGs) in plants grown in the dark. GO terms for biological processes (BP), cellular components (CC) and molecular functions (MF) are shown. (c) Pie chart showing the number and percentage of GLK target genes differentially expressed in the dark. (d) Venn diagrams showing the overlapping DEGs in plants grown under normal light and high‐light conditions. (e) Venn diagram showing the overlapping DEGs in plants grown in the dark and under normal light or high‐light conditions. (f) GO analysis of co‐regulated DEGs in the dark and under normal light or high‐light conditions shown in (e).

To investigate how OsEME1 regulates chloroplast development, we conducted RNA‐seq analysis of the *k48* mutant together with wild‐type KY131. We grew the plants under a long‐day photoperiod for 6 days and collected one batch of samples at the end of the dark period (designated as dark). We then exposed the seedlings to NL or HL conditions for 5 h before harvesting. We identified differentially expressed genes (DEGs) with a fold‐change of at least 1.5 and a false discovery rate (FDR) of 0.05 between *k48* and KY131 (Data S3). In the dark, 1445 genes showed altered expression, including 672 genes that were upregulated and 773 genes that were downregulated in *k48* compared to KY131 (Figure 3a and Data S2). Gene Ontology (GO) analysis revealed that the DEGs are involved in photosynthesis, salicylic acid biosynthesis, protein folding and phosphorylation, cell surface receptor signalling and nucleotide binding (Figure 3b and Data S1). The upregulated genes in *k48* are related to photosynthesis (chloroplast development), endoribonuclease activity and protein folding, while the downregulated genes in *k48* are related to response to hormones, protein kinase activity (protein phosphorylation) and nucleotide binding (catalytic activity and transferase activity) (Figure S7a and Data S1). Among the upregulated genes, 83 genes are related to chloroplast development, 17 of which were reported as the target genes of GLK1/2 in rice (Figure 3c), implying that OsEME1 regulates chloroplast development, the DNA damage signalling pathway and DNA‐binding activity.

Under NL conditions, 970 DEGs were identified in *k48* compared to KY131, including 671 upregulated and 299 downregulated genes. By contrast, under HL conditions, 3548 DEGs were identified in *k48*, including 1962 upregulated and 1586 downregulated genes (Figure 3a and Data S2), suggesting that HL greatly altered gene expression in *k48*. We compared the DEGs between NL and HL conditions: 496 genes (group ①) were upregulated under both NL and HL conditions, while 1466 genes (group ②) were upregulated in *k48* under HL; 161 genes (group ③) were downregulated under both conditions, whereas 1425 genes (group ④) were downregulated only under HL conditions (Figure 3d and Data S2). GO analysis showed that the genes upregulated under both conditions (group ①) are involved in DNA repair, DNA recombinase assembly, chromosome exchange activity and carbohydrate metabolism. Group ② genes are associated with proteasome‐mediated protein catabolic process, carboxylic acid metabolic process, nucleoside phosphate binding (catalytic activity and ligase activity) and oxidoreductase activity (Figure S7b and Data S1). Group ③ genes are associated with small molecule metabolic processes. Finally, group ④ genes are involved in chromosome remodelling, DNA replication, gene expression, the cell cycle and cell division (Figure S7c and Data S1).

We selected four DEGs to validate the RNA‐seq data by RT‐qPCR. Transcript accumulation for *PALE YELLOW GREEN 7* (*OsPYG7*, encoding a tetratricopeptide repeat domain‐containing protein), *CRYPTOCHROME 3* (*OsCRY3*), *SOUL HEME‐BINDING PROTEIN 4* (*OsSOUL4*) and *hypothetical chloroplast open reading frames 45–2* (*Osycf45‐2*) were lower in *k48* under NL but higher in the dark or under HL conditions compared to KY131, which is consistent with their expression patterns obtained by RNA‐seq (Figure S8a).

Next, we identified DEGs among the dark, NL and HL groups. The expression of 245 genes (group ⑤) was altered in *k48* under all 3 conditions and 260 genes were specifically affected under NL, whereas 2542 genes (group ⑥) were differentially expressed under HL conditions (Figure 3e and Data S2). GO analysis revealed that the group ⑤ genes were enriched in GO terms related to DNA DSB repair, DNA recombinase assembly and condensed nuclear chromosome (Figure 3f and Data S1), suggesting that OsEME1 plays a role in DNA repair. The group ⑥ genes are involved in chromosome condensation and gene expression, organelle organization, protein metabolic process and microtubule motor activity (Figure S8b and Data S1). These data indicate that the *k48* line is sensitive to high‐light irradiation and that OsEME1 is involved in DNA damage repair.

### Mutations in *OsEME1* increase sensitivity to DNA damage and lead to cell cycle arrest

**Figure 4:**
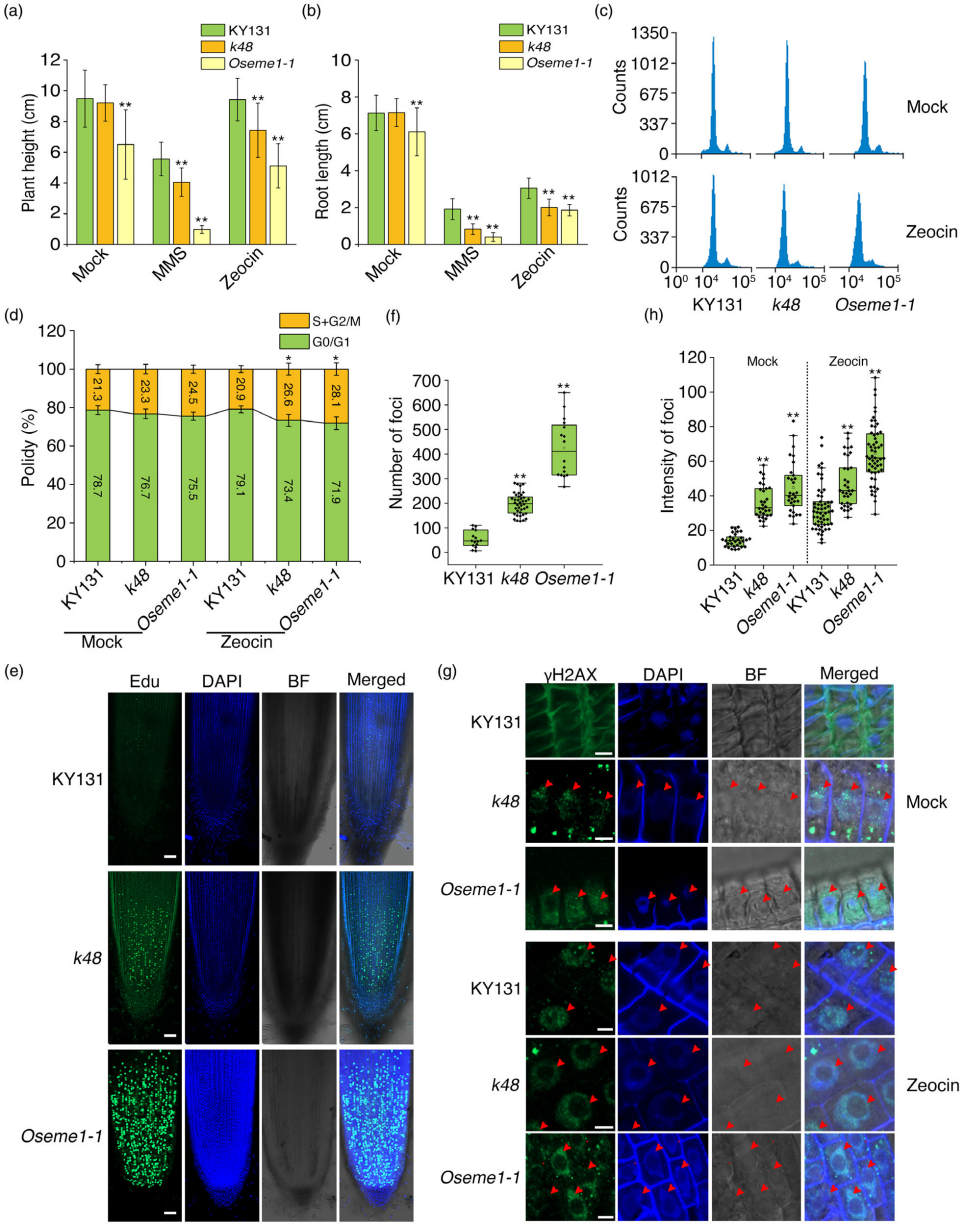
Loss of *OsEME1* increases sensitivity to DNA damage and cell cycle arrest. (a, b) Plant height (a) and root length (b) of seedlings grown in Hoagland's medium without (Mock) or with DNA‐damaging agents, 75 μg/mL methyl methanesulfonate (MMS) or 50 μg/mL Zeocin, for 5 days. (c, d) Analysis of nuclear DNA content (c) and nuclear ploidy (d) in the shoot apices of plants grown in Hoagland's medium without (Mock) or with 200 μg/mL Zeocin for 3 days. (e, f) EdU (5‐ethynyl‐2′‐deoxyuridine) fluorescence (e) and number of Edu‐GFP foci (f) in the roots of seedlings grown in Hoagland's medium for 3 days. More than 15 root tips were used for statistical analysis in (f). BF, bright field; DAPI staining shows nuclear signals. Bars, 50 μm. (g, h) Immunolocalization (g) and quantification of foci (h) of γH2AX in the roots of seedlings grown in Hoagland's medium without (Mock) or with 200 μg/mL Zeocin for 5 days. Red arrows indicate γH2AX foci in chromatin. More than 50 root cells were used for statistical analysis in (h). Bars, 4 μm. For (a, b, d, f and h), data are mean ± SD from three biological replicates. Asterisks indicate significant differences using Student's *t*‐test (**P < 0.01, *P < 0.05).

RNA‐seq and GO analysis suggested that OsEME1 might be involved in repairing DNA damage. To test this hypothesis, we grew *k48* and *Oseme1‐1* plants together with wild‐type KY131 in medium supplemented without (Mock) or with the DNA‐damaging reagent methyl methanesulfonate (MMS) or Zeocin for 5 days (Fulcher and Sablowski, 2009; Hartung *et al*., 2006). Under mock conditions, the *Oseme1‐1* mutant had slightly shorter shoots and roots than KY131 and *k48*. Strikingly, treatment with 75 μg/mL of MMS or 50 μg/mL of Zeocin strongly inhibited root and shoot growth, especially in the mutants. The plant height and root length were significantly reduced in *k48* and *Oseme1‐1* compared to KY131 under both conditions (Figure 4a,b and Figure S9). We then performed flow cytometry assays to analyse cell cycle progression in the stem apex. The DNA contents at the S and G_2_ stages following Zeocin treatment increased in *k48* and *Oseme1‐1* cells compared to KY131 (Figure 4c,d).

The 5‐ethynyl‐2′‐deoxyuridine (EdU) proliferation assay has been used to detect the S phase of the cell cycle in Arabidopsis and rice (Kotogány *et al*., 2010; Yoshiyama *et al*., 2017). To distinguish cells between the S and G_2_ stages, we performed EdU labelling of intact roots of germinating seedlings and determined that *k48* and *Oseme1‐1* plants had significantly more cells in the S phase than KY131 (Figure 4e,f). We then performed an immunofluorescence assay to identify γ2HAX foci, which are used as a measure of DNA repair activity in plant cells upon DNA damage (Cheng, 2013). The *k48* and *Oseme1‐1* mutants exhibited significantly more γ2HAX foci than KY131 with or without Zeocin treatment (Figure 4g,h). These results suggest that OsEME1 regulates DSB repair and the cell cycle.

### OsEME1 directly binds to and cleaves typical DNA‐break substrates

**Figure 5:**
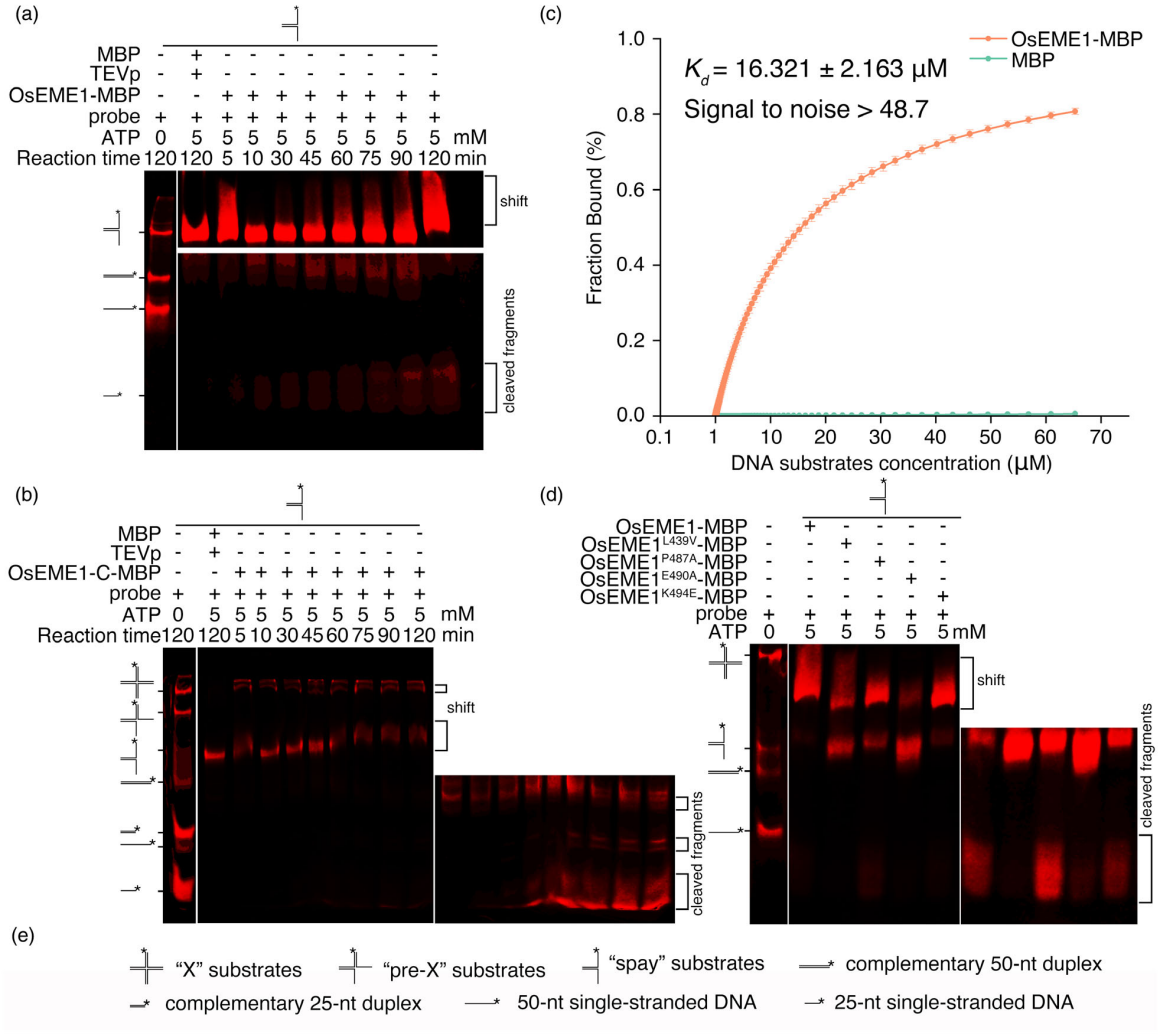
OsEME1 directly binds to and cleaves typical DNA‐break substrates. (a, b) Detection of the endonuclease activity of OsEME1 (a) and OsEME1‐C (b) on Y12 substrates. (c) Microscale thermophoresis (MST) analysis. Labelled DNA damage Y12 substrates were incubated with a series of concentrations of maltose binding protein (MBP) control and OsEME1‐MBP proteins and detected on a Monolith NT.115 apparatus. An MST‐on time of 10 s and 2% excitation power were used for analysis and an equilibrium dissociation constant (Kd) value was derived for the binding system. Data are mean ± SD from three biological replicates. (d) Detection of the endonuclease activity of OsEME1 point mutants on Y12 substrates. (e) Various DNA substrates used in (a), (b) and (d).

Since *OsEME1* is a homologue of *EME1*, which encodes an endonuclease, we measured the endonuclease activity of OsEME1 by performing a nuclease assay, followed by gel electrophoresis. We constructed the typical ERCC1‐XPF substrate Y12 by annealing two 50‐nt, PAGE‐purified oligonucleotides with 25‐nt compatible bases to its 5′ terminus and 25‐nt incompatible bases to its 3′ terminus (Figure S10b,d) (Boddy *et al*., 2001). We expressed and purified the recombinant proteins maltose binding protein (MBP)‐OsEME1 and MBP‐OsEME1‐C (a truncated C‐terminal fragment of OsEME1) and treated them with tobacco etch virus protease (TEVp) at 4 °C for 16 h to cleave the MBP tag (Figure S10a). We mixed the purified proteins with the reaction system and incubated them at 30 °C for up to 120 min. Major binding shifts were detected in the OsEME1 and OsEME1‐C lines but not in the MBP control (Figure 5a,b). The major cleavage products were obtained in OsEME1 and OsEME1‐C, and their levels gradually increased over time, while the levels of OsEME1‐C products increased compared to those of OsEME1 (Figure 5a,b), indicating that the C terminus of OsEME1 is responsible for its endonuclease activity. In a microscale thermophoretic assay (MST), OsEME1 showed higher affinity to the Y12 substrates (equilibrium dissociation constant [Kd] = 16.321 ± 2.163 μM) than did the MBP control (Figure 5c).

Four conserved residues in EME1 are critical for its endonuclease activity in mammalian cells (Chang *et al*., 2008; Gwon *et al*., 2014; Hua *et al*., 2022). We thus generated variants of OsEME1 protein based on a sequence comparison of OsEME1 with EME1 in *Homo sapiens* (OsEME1^L439V^, OsEME1^P487A^, OsEME1^E490A^ and OsEME1^K494E^) and performed an electrophoretic mobility shift assay (EMSA) using equal amounts of various proteins. The binding shifts and levels of cleavage products were dramatically reduced for OsEME1^L439V^, OsEME1^E490A^ and OsEME1^K494E^ compared to wild‐type OsEME1; the binding shift was decreased, but the level of cleavage products was increased for OsEME1^P487A^ compared to wild‐type OsEME1 (Figure 5d), suggesting that these four conserved amino acids are essential for the endonuclease activity of OsEME1.

### OsEME1 directly binds to and cleaves DNA breaks in *OsGLK1/2* via their duplex arms

RNA‐seq analysis indicated that many genes involved in regulating chloroplast development were upregulated in *k48* in the dark. OsGLK1 and OsGLK2 are nucleus‐encoded transcription factors that play important roles in chloroplast development (Tu *et al*., 2022; Wang *et al*., 2013). The expression levels of *OsGLK1* and *OsGLK2* were significantly lower in *k48* than in KY131 seedlings grown under NL conditions (Figure S10c). To investigate whether OsEME1 stabilizes chloroplast development by directly binding to target DNA breaks in genes and cleaving their duplex arms, we performed EMSA using *OsGLK1* and *OsGLK2*. We annealed 50‐nt oligonucleotides in the first exons of these genes with a partially matched oligonucleotide (25‐nt bases of 50‐nt oligonucleotides with a compatible 5′ terminus) to generate the specific Y12 substrates *OsGLK1‐Y12* and *OsGLK2‐Y12*. OsEME1 and OsEME1‐C bound to and caused cleavage shifts of *OsGLK1‐Y12* and *OsGLK2‐Y12* in a manner similar to Y12 (Figure S10e–h). These results suggest that OsEME1 helps maintain *OsGLK1/2* expression by cleaving their duplex arms at DNA breaks.

### OsEME1 interacts with OsMUS81

**Figure 6:**
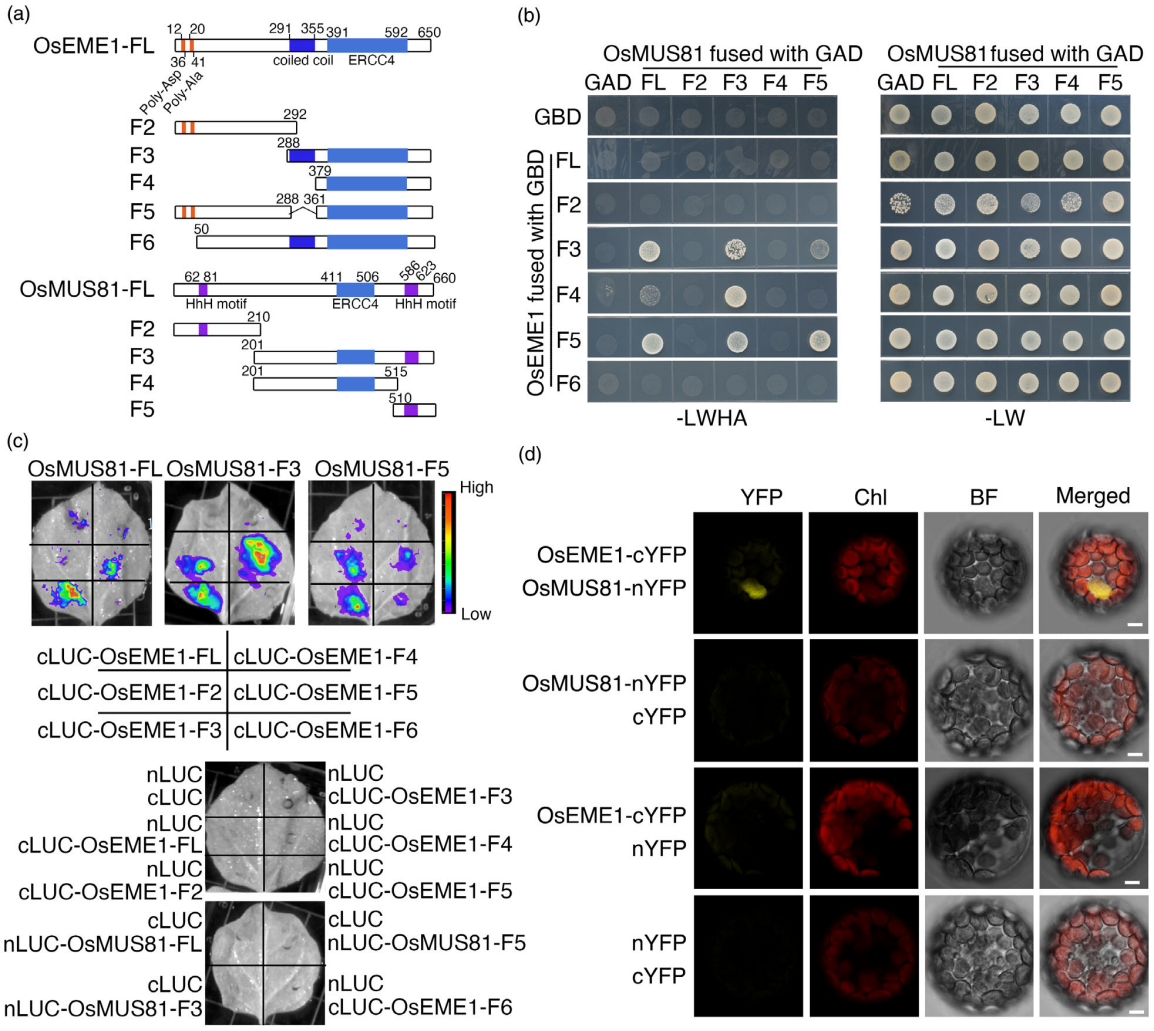
OsEME1 interacts with OsMUS81. (a) Diagram of OsEME1 and OsMUS81 and their truncations. The conserved domains/motifs and amino acid positions are shown. FL indicates full length of genes. (b) Yeast two‐hybrid assay. OsMUS81 and its truncated fragments were fused with the GAL4 activation domain (GAD). OsEME1 and its fragments were fused with the GAL4 DNA‐binding domain (GBD). –LW indicates medium lacking Leu and Trp; –LWHA indicates medium lacking Leu, Trp, His and Ade. (c) Luciferase complementation imaging (LCI) assay. OsMUS81 and its truncated fragments were fused with nLUC; OsEME1 and its fragments were fused with cLUC. All plasmids were expressed in *N. benthamiana* leaves, and the plants were incubated in the dark for 2 days. (d) BiFC assay. OsMUS81‐nYFP and OsEME1‐cYFP or control plasmids were co‐expressed in Arabidopsis protoplasts, and the protoplasts were incubated in the dark for 16 h. BF, bright field; Chl, chlorophyll autofluorescence. Bars, 5 μm.

Homologues of EME1 and MUS81 interact to form heterotetramers in mammals, yeasts and Arabidopsis (Boddy *et al*., 2001; Ciccia *et al*., 2003; Geuting *et al*., 2009; Oğrünç and Sancar, 2003). To investigate whether OsEME1 also interacts with OsMUS81 in rice, we truncated OsEME1 and OsMUS81 into different fragments based on their domain structures (Figure 6a). We fused the OsEME1 fragments with the GAL4 DNA‐binding domain (GBD) and the OsMUS81 fragments with the GAL4 activation domain (GAD). A yeast two‐hybrid assay revealed that the ERCC4 domain of OsEME1 (the F4 fragment) and the HhH motif of OsMUS81 (F5 fragment) are responsible for their interaction (Figure 6b).

We then performed a luciferase (LUC) complementation assay in *Nicotiana benthamiana* leaves. Co‐infiltration of OsMUS81‐nLUC and OsEME1‐F3/F5‐cLUC, OsMUS81‐F3‐nLUC and OsEME1‐F2/F3/F5‐cLUC and OsMUS81‐F5‐nLUC and OsEME1‐F2/F3/F5 constructs reconstituted LUC activity in the leaves, whereas co‐infiltration of the control plasmids did not (Figure 6c). In a bimolecular fluorescence complementation (BiFC) assay in Arabidopsis, co‐transformation with OsEME1 fused with the C‐terminal half of yellow fluorescent protein (OsEME1‐cYFP) and OsMUS81 fused with the N‐terminal half of YFP (OsMUS81‐nYFP), but not the empty vector controls, produced nucleus‐localized YFP signals in the protoplasts (Figure 6d). Collectively, these results confirm the notion that OsEME1 interacts with OsMUS81 and that the ERCC4 domain of OsEME1 is required for this interaction.

### OsMUS81 is not responsible for cleavage of X‐structure substrates

To examine the biological roles of *OsMUS81*, we used CRISPR/Cas9 to generate two *Osmus81* mutants. The *Osmus81‐1* mutant contained a 156‐bp deletion from the first intron to the second intron of this gene (Figure S11a). This mutant had reduced *OsMUS81* transcript levels and exhibited yellow leaves compared to KY131 (Figure S11b–d). The second mutant, *Osmus81‐2*, contained a one‐base insertion in editing site 1 and a two‐base deletion in editing site 2 (Figure S11a) and displayed reduced *OsMUS81* transcript levels. *Osmus81‐2* appeared light green compared to KY131 (Figure S11b–d).

**Figure 7:**
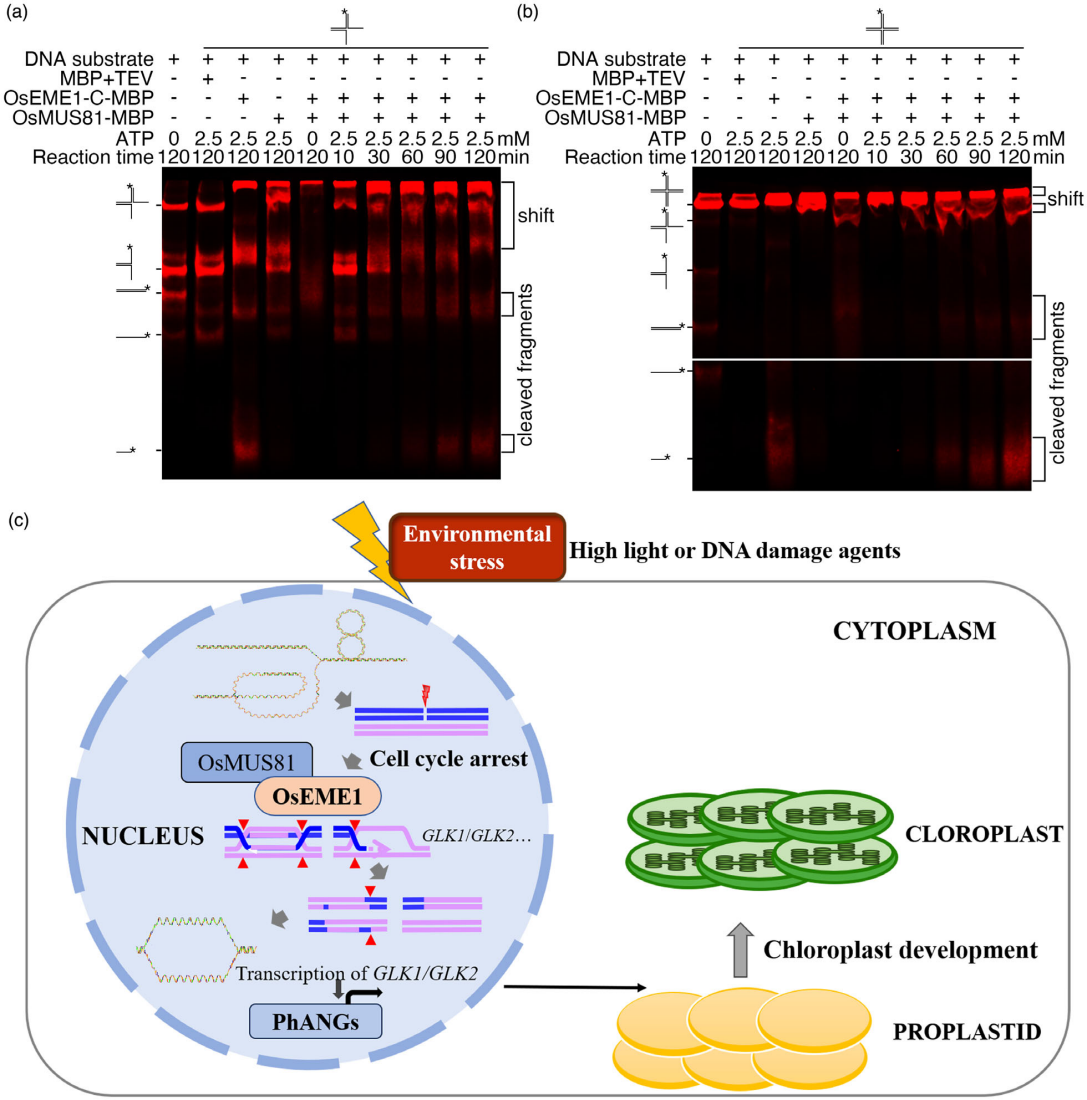
OsEME1 functions as an endonuclease during homologous recombination repair in rice. (a, b) Detection of endonuclease activity showing the cleavage of pre‐X substrates (a) or X substrates (b) by OsEME1‐C, OsMUS81 or the OsEME1‐C‐OsMUS81 complex. (c) A proposed working model of the role of OsEME1 in homologous recombination repair in rice. DNA double‐strand breaks appear in rice seedlings grown under high‐light conditions or in the presence of DNA‐damaging agents. The DNA damage response pathway is activated, leading to the repair of the damaged strands through the homologous recombination pathway and cell cycle arrest. Double Holiday junctions rapidly accumulate in the plants, and OsEME1 is recruited and directly binds to and cleaves the Holiday junction substrates, which helps maintain the stability of the genomic DNA and normal transcription, including *GLK1*/*GLK2* transcription during the seedling stage. As important chloroplast development transcription factors, *GLK1*/*GLK2* regulate the expression of many chloroplast development‐related genes, including photosynthesis‐associated nuclear genes and photosynthesis‐associated plastid genes.

The OsEME1–OsMUS81 interaction prompted us to explore whether their endonuclease activities act cooperatively. We performed an EMSA using interruptible DNA substrates [X12, with a 12‐bp region of homology in its centre, allowing the junction to migrate (Parsons *et al*., 1990)] derived from four oligonucleotides (or pre‐X12, derived from three oligonucleotides). We mixed OsEME1‐C‐OsMUS81 recombinant protein with DNA substrates and examined the binding shifts and cleavage shifts in the OsEME1‐C and OsEME1‐C‐MUS81 lanes in the gel. The production of cleaved products increased over time, but the product signals in OsEME1‐C resembled the signals in OsEME1‐C‐OsMUS81, even though binding shifts were observed in the OsMUS81 lane (Figure 7a,b). These data suggest that OsMUS81 binds to X‐structure substrates but is not responsible for the cleavage of junction DNA substrates.

## Discussion

In contrast to yeasts and mammals, many factors that function in DNA double‐strand damage repair in plants have not been explored. In this study, we revealed the biochemical function of OsEME1 as an endonuclease involved in DNA double‐strand damage repair in rice. OsEME1 regulates chloroplast development and root elongation in rice by maintaining the stability of the genome and transcription (Figure 7c).

### OsEME1 regulates chloroplast development and division by maintaining the transcription of chloroplast‐related genes and nuclear genome integrity

Light induces the expression of numerous chloroplast‐associated genes and the rapid replication and division of chloroplasts during the early stages of rice seedling development (López‐Juez *et al*., 2008). Here, the *k48* mutant showed reduced mRNA and protein levels of core photosystem subunits, decreased chlorophyll content and defective chloroplast structure (Figure 1 and Figure S1). RNA‐seq indicated that genes related to chloroplast development were downregulated in *k48* in the light and upregulated in the dark relative to KY131 (Figure S7a). The expression levels of *OsGLK1* and *OsGLK2* were significantly reduced in *k48* relative to KY131 under normal light conditions (Figure S10c). An EMSA revealed that OsEME1 binds to and cleaves *OsGLK1*/*OsGLK2* DNA substrates with specific structures (Figure S10e–h). We suggest that OsEME1 cleaves damaged double‐stranded DNA substrates such as *OsGLK1* and *OsGLK2* DNA and maintains gene transcription. Although most genes that function in chloroplast development or photosynthesis are differentially expressed when a seedling emerges from the soil until the completion of leaf development (Shi *et al*., 2022), OsEME1 maintains the transcription of numerous genes related to chloroplast development during these stages.

Several genes that maintain the stability of the chloroplast genome have been identified in Arabidopsis. WHY proteins have specific binding activity to single‐stranded DNA and maintain chloroplast genome stability by stabilizing single‐stranded DNA and guiding it through conservative repair mechanisms (Cappadocia *et al*., 2010; Maréchal *et al*., 2009). DNA polymerase I B (PolI B) mediates chloroplast genome replication and damage repair (Parent *et al*., 2011). RECA1/WHY1/WHY3 mediate the cell cycle via SOG1/SMR5/SMR7 and thus regulate chloroplast genome stability (Duan *et al*., 2020; Lepage *et al*., 2013; Zampini *et al*., 2015). *OsEME1* mutants exhibit leaf variegation and a large amount of γH2AX in the nucleus. Whether OsEME1 is directly involved in maintaining the stability of the chloroplast genome remains to be further investigated.

Chloroplasts are important environmental sensors that perceive signals from the nucleus and regulate plant growth and development via feedback regulation. Instability of the chloroplast genome induces ROS accumulation in the chloroplast. ROS signals from chloroplasts to the nucleus activate SOG1 to regulate the cell cycle (Duan *et al*., 2020). *k48* exhibited severe leaf striping after high‐light treatment (Figure 1h,i). Genes related to oxidoreductase activity, phagophore assembly, proteasome regulatory particle and proteasome‐mediated ubiquitin‐dependent protein catabolic process were upregulated, whereas genes related to DNA replication and gene expression, the cell cycle and organelle fission were downregulated in rice plants transferred to HL conditions (Figure S7b,c), revealing that the deficiency of OsEME1 causes oxidative‐stress‐induced damage to rice chloroplasts, which further increases after HL treatment. In addition to removing HR repair substrates in the nucleus, OsEME1 might coordinate the movement of oxidative signals from the chloroplast to the nucleus, thus boosting plant growth and development.

### OsEME1 is essential for responses to DSBs and functions in HR repair

Besides showing leaf striping, *k48* and *Oseme1‐1* seedlings were hypersensitive to environmental stimuli and DNA‐damaging agents. Leaf defects were exacerbated when the plants were exposed to HL conditions (Figure 1h–k); plant height and root length were significantly reduced in *k48* and *Oseme1‐1* compared to KY131 after treatment with DNA‐damaging agents (MMS and Zeocin) (Figure 4a,b and Figure S9). A large number of stagnant (S phase) cells accumulated in *k48* and *Oseme1‐1* root tip cells (Figure 4e,f) and the proportion of cells in the S and G2 phases significantly increased in both *k48* and *Oseme1‐1* after treatment with Zeocin (Figure 4c,d). Fluorescent signals from the DNA double‐strand damage marker protein γH2AX were observed in *k48* and *Oseme1‐1* root tips, which were enhanced after Zeocin treatment (Figure 4g,h). RNA‐seq showed that the expression of genes associated with double‐strand damage repair was activated in *k48* (Figure 3f and Figure S7a). These results suggest that OsEME1 plays an important role in mediating DSB repair, the release of cell cycle arrest and plant growth and development.

In yeasts, mammals and Arabidopsis, EME1 forms heterotetramers with MUS81, binds to Holliday junctions generated after HR repair and introduces incisions in duplex DNA 5′ of a double‐strand–single‐strand junction (Abraham *et al*., 2003; Boddy *et al*., 2001; Ciccia *et al*., 2003; Geuting *et al*., 2009; Oğrünç and Sancar, 2003). In an endonuclease activity assay, we observed cleaved products in lanes where OsEME1 protein was added (Figure 5), indicating that OsEME1 possesses DNA cleavage activity. Compared to the full‐length OsEME1, the C terminus of OsEME1 (OsEME1‐C) cleaved the substrates more efficiently (Figure 5a,b), indicating that the C terminus is the functional region of cleavage activity. The C terminus of OsEME1 possesses the ERCC4 domain, which is conserved in several species and shows nucleic acid binding activity (Ciccia *et al*., 2008). The binding‐shift and product‐shift signals were weakened in lanes containing OsEME1^L439V^, OsEME1^E490A^ and OsEME1^K494E^, and the product shift was enhanced in the lane containing OsEME1^P487A^ (Figure 5d), demonstrating the importance of the four conserved amino acid sites for the enzymatic activity of OsEME1. Similar to its homologues in mammals and yeasts, OsEME1 interacted with OsMUS81 (Figure 6) and cleaved the pre‐X or X DNA substrates *in vitro* (Figure 7a,b).

In mammals and Arabidopsis, WEE1 directly phosphorylates CDKs and thus inhibits the activities of cell cycle proteins (Ghelli Luserna di Rorà *et al*., 2020; Jin *et al*., 2020; Shimotohno *et al*., 2006), resulting in cell cycle arrest in the S phase and leading to DNA replication. Wee1 inhibits the activity of the Mus81–Eme1 complex through direct interactions with Mus81 in 293T cells, thereby allowing cells in the S phase to undergo DNA replication (Domínguez‐Kelly *et al*., 2011). In Arabidopsis, WEE1 directly phosphorylates FBL17 (F‐BOX LIKE17, the ubiquitination ligase of the CDK repressor KRP6/KRP7), which mediates its ubiquitination‐mediated degradation and thus blocks cell cycle progression (Pan *et al*., 2021, 2023). F‐box proteins are components of the SCF (S phase kinase‐associated protein, Cullin, F‐box containing) E3 ligase complex and several F‐box proteins mediate chloroplast‐to‐nucleus ROS signalling. SLAC7 (SLOW ANION CHANNEL‐ASSOCIATED 7), CPR1 (CONSTITUTIVE EXPRESSOR OF PATHOGENESIS RELATED GENES 1) and Cucurbit Chlorotic Yellows Virus p22 protein are related to defence against signalling and protect chloroplasts from stress (Chen *et al*., 2019; Fan *et al*., 2015; Hedtmann *et al*., 2017). ORESARA 9 (ORE9) and FBK12 (F‐BOX PROTEIN CONTAINING A KELCH REPEAT MOTIF 12) mediate leaf senescence in Arabidopsis and rice (Chen *et al*., 2013; Woo *et al*., 2001). Investigating the relationships between EME1, WEE1 and F‐box proteins in rice is important for understanding double‐stranded DNA damage signalling and the protection of chloroplasts from stress.

In summary, we discovered a biochemical role for OsEME1 in HR repair and a biological role for this protein in chloroplast development. The MUS81–EME complex directly binds to and cleaves typical DNA substrates produced after HR repair, a process conserved in yeasts, mammals, dicots and monocots. Surprisingly, rice contains only a single *EME* gene. OsEME1 regulates chloroplast development and reproductive growth. OsEME1 is involved in chloroplast division and cell cycle arrest and might coordinate the movement of oxidative signals from the chloroplast to the nucleus.

## Materials and methods

### Plant materials and growth conditions

Kongyu 131 (KY131, *Oryza sativa* L. *japonica*) is an early‐maturity *japonica* rice variety grown in Heilongjiang province, China (Zhou *et al*., 1997). KY131 was mutagenized with EMS and *k48*, a loss‐of‐function mutant that exhibited striped albino leaves, was identified. Mutants were generated by CRISPR/Cas9‐mediated gene editing in the KY131 background as previously described (Ma and Liu, 2016). The binary constructs (described below) were introduced into *Agrobacterium tumefaciens* strain EHA105 by electroporation and introduced into wild‐type KY131. Transgenic plants were selected on Murashige and Skoog (MS) plates containing 50 mg/L hygromycin. The mutation sites were verified by PCR and sequencing. Primers used to detect mutations are listed in Table S1. Homozygous lines were used in all experiments. For experiments using seedlings, plants were grown under normal light (NL) intensity (~300 μmol/m^2^/s), long‐day (16 h light/8 h dark) conditions in a growth chamber at 28 °C for 4–12 days (normal conditions). For HL conditions, plants were grown under normal light conditions for 9 days, transferred to continuous high light intensity (700 μmol/m^2^/s) at 28 °C, and grown for 3 days. Light was supplied by cool fluorescent lamps.

For DNA damage treatments, KY131, *k48* and *Oseme1* plants were grown in Hoagland's medium with or without different DNA damage regents (Zeocin as a glycopeptide antibiotic induces DNA double‐strand breaks to genome, while MMS induces damage through alkylation of DNA bases) (Adachi *et al*., 2011; Berdy, 1980; Fulcher and Sablowski, 2009) for 6 days and physiological parameters were recorded. For the immunofluorescence assay and the EdU assay, seedlings were cultivated in Hoagland's medium with or without Zeocin for 3 or 5 days, and the main roots were harvested for analysis.

### Analysis of pigment contents

Samples were collected from 12‐day‐old rice seedlings and ground to a powder in liquid nitrogen. Chlorophylls and carotenoids were extracted as previously described (Du *et al*., 2021). The amounts of these pigments were calculated using the equations: chlorophyll a (Chl a) = 13.95 × D_665_–6.88 × D_649_; Chl b = 24.96 × D_649_–7.32 × D_665_; carotenoid (Car) = (1000 × D_470_–2.05 × Chl a – 114 × Chl b)/245 (Porra *et al*., 1989).

### Transmission electron microscopy

The third leaves were collected from 12‐day‐old rice seedlings, dehydrated and embedded as previously described (Liu *et al*., 2012). The leaves were cut into 0.25‐cm^2^ squares and fixed at 4 °C in 2.5% glutaraldehyde in 0.1 M phosphate‐buffered saline (PBS, pH 7.2) overnight. The leaf squares were rinsed three times with 0.1 M PBS for 30 min, postfixed in 2% OsO_4_ in 0.1 M PBS at 4 °C for 4 h, rinsed three times with 0.1 M PBS and dehydrated in the following ethanol series: 30%, 50%, 70%, 90% and 100%. The leaf squares were transferred successively to 3:1, 2:1, 1:1 and 1:2 (v/v) ethanol/acetone, followed by acetone. Subsequently, the leaf squares were transferred to 3:1, 1:1 and 1:3 (v/v) acetone/Epon 812, followed by Epon 812. The samples were embedded in Epon 812 and polymerized at 60 °C. Ultrathin sections were cut using a Leica Ultracut R ultramicrotome and collected on copper grids. The sections were stained with 1% uranyl acetate and lead citrate and observed under a transmission electron microscope (HT7700, HITACHI, Japan).

### RT‐qPCR

Total RNA was extracted from the samples using an RNA Extraction kit (Accurate Biology, China) and RNA concentration was determined using a Nanodrop 2000 spectrophotometer (Thermo Fisher Scientific). First‐strand cDNA was transcribed from equal amounts of RNA templates with reverse transcriptase (Invitrogen). Quantitative PCR was performed using the cDNA samples and a SYBR Premix Ex‐Taq Kit (Takara) in a LightCycler 480 instrument (Roche) following the manufacturer's instructions. Expression levels were calculated using the 2^−ΔΔCT^ method from three biological replicates. Relative expression levels were normalized to the levels of the *OsUBQ5* control. PCR primers are shown in Table S1.

### Plasmid construction

The full‐length open reading frames of *OsEME1* and *OsMUS81* were amplified from first‐strand cDNA obtained from wild‐type KY131 and cloned into the pEASY‐Blunt vector (TransGen). To facilitate subsequent cloning, one MfeI restriction site within the *OsEME1* open reading frame was mutagenized without changing the encoded amino acid sequence by site‐directed mutagenesis (TransGen). The resulting DNAs were cloned into the pEASY vector to generate pEASY‐OsEME1m. To generate a series of deletions, the full length (FL), F2, F3, F4, F5 and F6 fragments of *OsEME1* and the FL, F2, F3, F4 and F5 fragments of *OsMUS81* were amplified using the pEASY‐OsEME1m plasmid or the pEASY‐OsMUS81 plasmid as the template and the corresponding primer pairs and cloned into the pEASY vector, resulting in pEASY‐FL/F2/F3/F4/F5/F6 (FL/F2/F3/F4/F5), respectively. To generate four point mutation derivatives within the OsEME1 fragment (OsEME1^L439V^, OsEME1^P487A^, OsEME1^E490A^ and OsEME1^K494E^), site‐directed mutagenesis was performed using pEASY‐EME1m as the template. The resulting vectors were named pEASY‐OsEME1^L439V^, pEASY‐OsEME1^P487A^, pEASY‐OsEME1^E490A^ and pEASY‐OsEME1^K494E^, respectively. All clones were validated by sequencing. Primers are listed in Table S1.

To generate constructs for the yeast two‐hybrid assay, the FL and F2/F3/F4/F5/F6 fragments of *OsEME1* were released from the corresponding pEASY plasmids by digestion with *Mfe*I and *Xho*I and ligated into the *Eco*RI–*Sal*I sites of the pGBKT7 vector (Clontech) to produce GBD‐OsEME1‐FL/F2/F3/F4/F5/F6, respectively. The FL and F2/F3/F4/F5 fragments of *OsMUS81* were cloned into the EcoRI–XhoI sites of the pGADT7 vector (Clontech) using a Seamless Assembly Cloning kit (CloneSmarter) to generate GAD‐OsMUS81‐FL/F2/F3/F4/F5, respectively.

To generate constructs for recombinant protein expression, pEASY‐OsEME1, pEASY‐OsEME1‐C and pEASY‐OsMUS81 were digested with *Kpn*I and *Xho*I, and the resulting fragments were inserted into the pMAL‐c5X vectors (Takara) with the same cohesive terminus, generating MBP‐OsEME1, MBP‐OsEME1‐C and MBP‐OsMUS81, respectively. For EMSA and the MST assay, the TEVp sequence was cloned into pMAL‐c5x‐OsEME1 (or pMAL‐c5x‐OsEME1‐C, pMAL‐c5x‐OsMUS81) via amplification to generate MBP‐TEVp‐OsEME1 (or MBP‐TEVp‐OsEME1‐C, MBP‐TEVp‐OsMUS81).

To prepare constructs for the luciferase complementation imaging assay, the FL and F2/F3/F4/F5/F6 fragments of *OsEME1* were released from the corresponding pEASY plasmids by digestion with *Kpn*I and *Xho*I and ligated into the *Kpn*I–*Sal*I sites of p1300‐cLUC (Chen *et al*., 2008). The FL and F3/F5 fragments of *OsMUS81* were released from the corresponding pEASY plasmids (digested with *Kpn*I and *Xho*I) and cloned into the *Kpn*I–*Sal*I sites of p1300‐nLUC.

To prepare constructs for the BiFC assay, full‐length *OsMUS81* and *OsEME1* were inserted into pSAT4A‐nYFP or pSAT4A‐cYFP to generate OsMUS81‐nYFP and OsEME1‐cYFP, respectively, using a Seamless Assembly Cloning kit (CloneSmarter). For subcellular localization of GFP‐OsEME1, the full‐length coding sequence of *OsEME1* was digested with *Kpn*I and *Bam*HI, and the resulting fragment was inserted into the pSAT6‐C1 vector.

### Yeast two‐hybrid assay

Yeast transformation, selection and two‐hybrid experiments were performed according to the Yeast Protocol Handbook (Clontech). Briefly, fusion proteins containing the GAL4 DNA‐binding domain and the GAL4 activation domain were co‐transformed into yeast strain Y2H Gold (Clontech) and selected on SD/‐Trp‐Leu plates. The positive colonies were transferred to SD/‐Trp‐Leu and SD/‐Trp‐Leu‐His‐Ade dropout plates. Growth on SD/‐Trp‐Leu‐His‐Ade medium indicates protein–protein interactions.

### Luciferase complementation imaging (LCI) assay

The nLUC/cLUC‐fusion constructs or conjugative p19 plasmids were introduced individually into *Agrobacterium* strain GV3101. An LCI assay was performed as previously described (Chen *et al*., 2008). After 2–3 days of transformation, the leaves were sprayed with d‐luciferin (2 μM, dissolved in 0.02% [v/v] Triton X‐100) and interactions were monitored using a NightSHADE LB985 Plant Imaging system (Berthold, Bad Wildbad, Germany) equipped with a charge‐coupled device camera. The experiments were repeated at least three times.

### Protein extraction and immunoblot analysis

Total proteins and chloroplast proteins were extracted from plants as previously described (Wang *et al*., 2020; Yuan *et al*., 2022). Samples were boiled in 1 × SDS loading buffer (10×, 125 mM Tris–HCl pH 6.8, 12% [w/v] SDS, 10% [v/v] glycerol, 22% [v/v] β‐mercaptoethanol, 0.001% [w/v] bromophenol blue) for 10 min and 10‐microgram protein samples were separated on a 10% (w/v, for total protein) or 12% (for chloroplast protein) SDS‐PAGE gel. Proteins were then transferred onto polyvinylidene fluoride membranes (Amersham), blocked with skim milk and immunoblotted with anti‐D1, anti‐psbO, anti‐CF_1_, anti‐cytf, anti‐RbcS, anti‐CP47, anti‐GFP or anti‐actin primary antibodies, followed by incubation with horseradish peroxidase‐conjugated secondary antibody. The signals were captured with a Chemiluminescence Imaging System (Biostep, Burkhardtsdorf, Germany).

### EdU assay

The EdU assay was performed using a Click‐iT EdU Alexa Fluor 488 Imaging kit (Invitrogen) as previously described (Zhang *et al*., 2021). Briefly, 3‐day‐old seedlings were submerged in 100 μM EdU (Invitrogen, Click‐iT EdU Alexa Fluor 488 HCS assay, Cat# C10350, Component A) for 60 min. Following incubation, the root tips were fixed with Immunohistochemical Fixative (4% [w/v] paraformaldehyde solution, P4500, Lablead, China) for 2 h at room temperature and washed three times with PBS buffer for 10 min each time. The root tips were incubated in EdU Detection Cocktail (Invitrogen, Click‐iT EdU Alexa Fluor 488 HCS assay, Cat# C10350) for 45 min, washed once in reaction rinse buffer (Invitrogen, Click‐iT EdU Alexa Fluor 488 HCS assay, Cat# C10350, Component F) and washed twice with PBS‐DAPI (5‐min washes with PBS containing 100 ng/mL DAPI). The EdU‐stained root tips were observed under a Zeiss LSM 980 confocal microscope (settings: GFP, excitation 488 nm, emission 498–604 nm; DAPI, excitation 405 nm, emission 409–480 nm).

### Flow cytometry

Flow cytometry was conducted with a flow cytometer (MoFlo‐XDP, Beckman) using 3‐day‐old buds of KY131, *k48* and *Oseme1‐1* seedlings following the manufacturer's instructions. Briefly, 3‐day‐old bud samples were finely chopped and incubated in 0.5 mL Galbraith's buffer (45 mM MgCl_2_, 20 mM MOPS, 30 mM sodium citrate, 0.1% [v/v] Triton X‐100, pH 7.0 and filtered through a 0.22‐μm filter) on ice (Dolezel *et al*., 2007). Cells were collected by gentle pipetting and filtered through a 400‐mesh nylon strainer. The samples were stained with 100 μg/mL PI simultaneously with 100 μg/mL RNase on ice for 10 min before the analysis using a MoFlo‐XDP flow cytometer and the software Summit v.5.2.

### BN‐PAGE

BN‐PAGE was performed as described (Flores‐Pérez and Jarvis, 2017; Li *et al*., 2022a) with some modification. Ten‐day‐old rice leaves were harvested and ground in cold CIB buffer (0.33 M sorbitol, 5 mM MgCl_2_, 5 mM EDTA, 5 mM EGTA, 20 mM HEPES‐KOH pH 8.0, 10 mM NaHCO_3_) on ice. The homogenates were filtered through two layers of Miracloth (Millipore) into a 50‐mL tube and centrifuged at 3000 ***g*** for 10 min at 4 °C. The pellets were washed once in CIB buffer and suspended in resuspension buffer (25 mM HEPES‐KOH, pH 8.0, complete protease inhibitor cocktail) at 1.0 mg chlorophyll/mL. An equal volume of resuspension buffer containing 2% (w/v) n‐dodecyl‐β‐D‐maltoside was added to the thylakoid suspension in a dropwise manner. Following incubation on ice for 10 min, insoluble material was removed by centrifugation at 20 000 ***g*** for 30 min. After centrifugation, the native proteins were loaded onto a 4%–13.5% gradient native PAGE gel and subjected to electrophoresis.

### Immunofluorescence assay

Immunofluorescence of chromosome squashes was detected as described (Cheng, 2013). Briefly, the root tips of 5‐day‐old rice seedlings (grown in modified Hoagland's medium with or without 200 μg/mL Zeocin) were fixed in 4% (w/v) paraformaldehyde for 24 h at 4 °C. The fixed materials were washed three times with PBS and squashed in PBS to generate slides. Each slide was frozen in liquid nitrogen, the coverslip was removed, and the sample on the slide was dehydrated through an ethanol series (70%, 90% and 100%) prior to immunostaining. The slides were incubated in a humidity chamber at 37 °C for 3 h with γH2AX antibody (Abcam, ab81299) diluted 1:500 in TNB buffer (0.1 M Tris–HCl, pH 7.5, 0.15 M NaCl and 0.5% blocking reagent). After three rounds of washing in PBS, a GFP‐conjugated goat anti‐rabbit antibody (1:1000; Thermo Fisher, 35 552) was added to the slides. After 1 h of incubation in a humidity chamber at 37 °C, the slides were washed three times with PBS and incubated in PBS containing 1 μg/mL DAPI. Images of the slides were captured under a Zeiss 980 fluorescence microscope (settings: GFP, excitation 488 nm, emission 498–604 nm; DAPI, excitation 405 nm, emission 409–480 nm).

### Recombinant protein expression and purification

The MBP‐TEVp‐OsEME1 (OsEME1‐C and OsMUS81) recombinant protein expression vectors were constructed and transferred into *Escherichia coli* strain BL21 (DE3) cells. For protein purification, the cells were resuspended in cold buffer A (25 mM Tris–HCl, pH 7.5, 150 mM NaCl and 1 mM dithiothreitol [DTT]), disrupted by sonication and centrifuged. The maltose binding protein (MBP)‐fused proteins were purified using Dextrin Sepharose (GE Healthcare, Boston, MA) according to the manufacturer's instructions. The affinity‐purified recombinant proteins MBP‐OsEME1‐C and MBP‐OsMUS81 (but not MBP‐OsEME1, which would be degraded during purification) were further purified by size‐exclusion chromatography using a HiLoad 16/60 Superdex 200 column (GE Healthcare) in buffer A (25 mM Tris–HCl, pH 7.5, 150 mM NaCl and 1 mM dithiothreitol [DTT]). The monomer or dimer peak fractions were collected and subjected to EMSA, and the MBP peak of MBP‐EME1‐C was collected and used for the control in the EMSA. All proteins were treated with TEVp and placed in endonuclease assay buffer for subsequent experiments.

### EMSA

#### Preparation of DNA substrates

DNA substrates were annealed as described (Boddy *et al*., 2001). All substrates were prepared by annealing with the appropriate combinations of the respective oligonucleotides and stored in TE buffer (10 mM Tris–HCl, pH 8.0, 10 mM EDTA, 50 mM NaCl). The oligonucleotide sequences were described previously (Boddy *et al*., 2001; Gaillard *et al*., 2003). Y12 substrates consisted of labelled oligonucleotide X1 and cold oligonucleotide X4; pre‐X12 substrates consisted of labelled oligonucleotide X1 and cold oligonucleotides X2 and X4; X12 substrates consisted of labelled oligonucleotide X1 and cold oligonucleotides X2, X3 and X4 (Figure S9d). Fragments (50‐nt long) of *OsGLK1* (LOC_Os06g24070) and *OsGLK2* (LOC_Os01g13740) containing the first exon were used to prepare the respective Y12 substrates.

#### Endonuclease assay

The *Escherichia coli* strain BL21 (DE3) cells were sonicated, and OsEME1‐MBP or OsEME1‐C‐MBP (with a TEVp site) was purified from the clarified lysate using MBP‐tagged protein purification resin. Following treatment with TEVp at 4 °C for 24 h, the eluates were placed in reaction buffer (25 mM Tris–HCl pH 7.5, 1 mM MgCl_2_, 100 mg/mL BSA and 1 mM DTT). As a control, MBP and TEVp also were placed in the reaction buffer. The reaction system (20 μL) contained 2.5 μM cyanine 5 (Cy5)‐labelled substrates in reaction buffer and 5 mM ATP. The reactions were performed at 30 °C by mixing the protein with the other reaction components. Cleavage reactions with the Y12 substrates (random oligonucleotides or specific target oligonucleotides such as *OsGLK1* or *OsGLK2*) contained 20 μM purified OsEME1 or OsEME1‐C protein or control MBP and TEVp proteins, respectively. The same concentration of OsEME1‐C (and OsMUS81) was used for pre‐X12 substrates and X12 substrates. For time‐course experiments, eight 20‐μL reaction systems were prepared and removed at time points 10, 30, 60, 90 and 120 min (for pre‐X12 and X12 substrates) or 10, 30, 45, 60, 75, 90 and 120 min for Y12 substrates. The reaction was stopped by adding one‐third volume of stop solution (50 mM EDTA, 0.6% SDS, 20% glycerol, 0.1% xylene cyanol FF and 0.1% bromophenol blue). A 20‐μL aliquot of each sample was run on a 15% TBE‐PAGE gel with DNA substrates used as a marker.

#### Fluorescence detection

The gel was scanned with an Odyssey XF system (LI‐COR) to detect the Cy5 fluorescent signal (excitation at 685 nm and emission collected at 720 nm).

### MST

Microscale thermophoretics were conducted using a Monolith NT.115 apparatus (NanoTemper Technologies, Germany). OsEME1‐MBP or MBP was purified and centrifuged at 15 000 ***g*** at 4 °C for 30 min. The supernatant was filtered through a 0.22‐μm membrane filter. To measure K values, 100 nM Cy5‐labelled Y‐Flag DNA substrates were incubated with a serial dilution of OsEME1‐MBP or MBP from 70 μM to 1.06 nM. A 10‐μL sample was loaded into each capillary and inserted into the MST instrument loading tray (Monolith NT.115). The thermophoresis experiments were carried out using 2% MST power and medium LED power at 30 °C.

### BiFC assay

The OsMUS81‐nYFP, OsEME1‐cYFP and control plasmids were introduced into Arabidopsis protoplasts as described (Li *et al*., 2022b). After 16 h of incubation at 22 °C, the YFP signal was observed under a ZEISS LSM 980 microscope with excitation at 514 nm and emission collected at 489–604 nm. Chlorophyll autofluorescence was excited at 514 nm and emission was collected at 650–750 nm.

## Author contributions

Y.D. carried out the majority of the experiments and wrote the paper; Y.L. performed phenotypic analysis of plants treated with DNA‐damaging agents, recombinant protein expression and purification, and GO analysis and discussed the project; W.T. constructed rice mutant pools and performed BSA sequencing of *k48* and KY131; W.M. and T.M. discussed and commented on the project; R.L. initiated and directed the study, and Y.D. and R.L. wrote the paper.

## Conflict of interest

The authors declare no competing financial interests.

## Supporting information


**Dataset S1** GO analysis information.

**Dataset S2** List of genes used for Venn analysis.

**Dataset S3** RNA‐seq data of the DEGs.

**Figure S1** Chloroplast autophagy in *k48* and analysis of chloroplast gene expression and protein accumulation in *k48* and KY131 seedlings.
**Figure S2** Cloning of *OsEME1*.
**Figure S3** Intron retention and premature translation termination of *OsEME1* in *k48*.
**Figure S4** Generation of *OsEME1* CRISPR/Cas9 lines.
**Figure S5** Sequence and expression analysis of *OsEME1*.
**Figure S6** Phylogenetic tree of EME1.
**Figure S7** Gene Ontology (GO) analysis of differentially expressed genes (DEGs) in *k48* grown in the dark or under light.
**Figure S8** Gene Ontology (GO) and gene expression analyses.
**Figure S9** Loss of *OsEME1* increases sensitivity to DNA damage.
**Figure S10** OsEME1 directly binds to and cleaves *OsGLKs* Y12 substrates.
**Figure S11** Generation of *Osmus81* CRISPR/Cas9 lines.
**Table S1** List of primers used in this study.

## Data Availability

The data that supports the findings of this study are available in the supplementary material of this article.
