# Supplementary material for: ESSENTIAL MEIOTIC ENDONUCLEASE 1 is required for chloroplast development and DNA repair in rice
Source: Plant Biotechnol J. 2025 May 7;23(7):2931–48. doi: 10.1111/pbi.70101 (PMC12205872; doi:10.1111/pbi.70101)
Supplement: Supplementary file 4 — Figure S1 Chloroplast autophagy in k48 and analysis of chloroplast gene expression and protein accumulation in k48 and KY131 seedlings. Figure S2 Cloning of OsEME1. Figure S3 Intron retention and premature translation termination of OsEME1 in k48. Figure S4 Generation of OsEME1 CRISPR/Cas9 lines. Figure S5 Sequence and expression analysis of OsEME1. Figure S6 Phylogenetic tree of EME1. Figure S7 Gene Ontology (GO) analysis of differentially expressed genes (DEGs) in k48 grown in the dark or under light. Figure S8 Gene Ontology (GO) and gene expression analyses. Figure S9 Loss of OsEME1 increases sensitivity to DNA damage. Figure S10 OsEME1 directly binds to and cleaves OsGLKs Y12 substrates. Figure S11 Generation of Osmus81 CRISPR/Cas9 lines. Table S1 List of primers used in this study. [file PBI-23-2931-s003.pdf]

Supplemental data

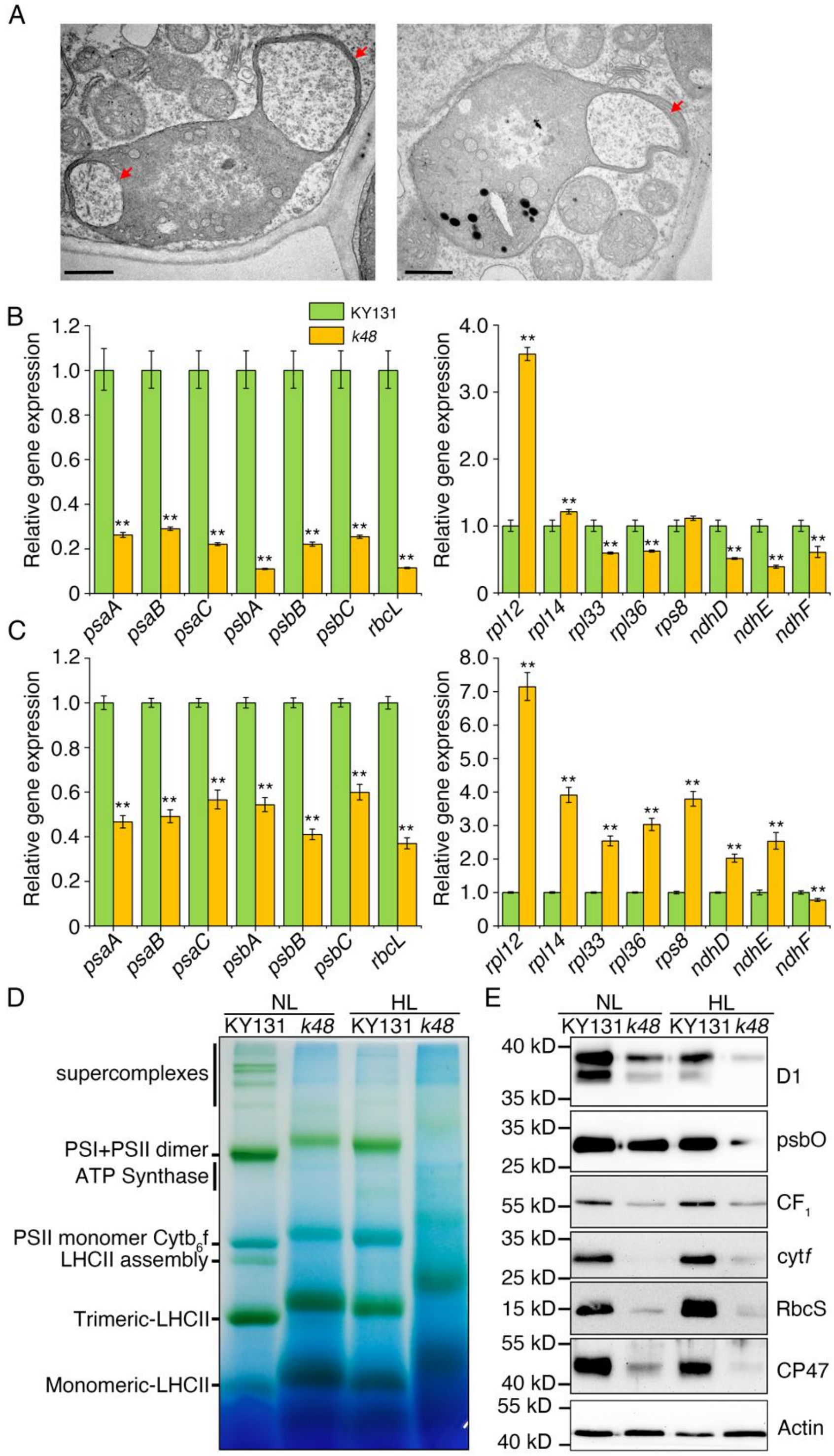

Figure S1. Chloroplast autophagy in *k48* and analysis of chloroplast gene expression and protein accumulation in *k48* and KY131 seedlings.

(A) Transmission electron microscopy of *k48* chloroplasts. Red arrows indicate chloroplast autophagy. Bars, 0.5 μm. (B and C) Expression analysis of plastid-encoded

RNA polymerase related- or nucleus-encoded plastid RNA polymerase related- genes in wild-type KY131 and *k48* plants grown under normal-light (NL) conditions (B) or high-light (HL) conditions (C), as shown in Figure 1. Data are mean ± s.d. from three biological replicates. Asterisks indicate significant differences using Student's *t*-test (** $P < 0.01$). (D) Blue native gel electrophoresis of thylakoid membrane protein complexes. (E) Immunoblotting of chloroplast proteins.

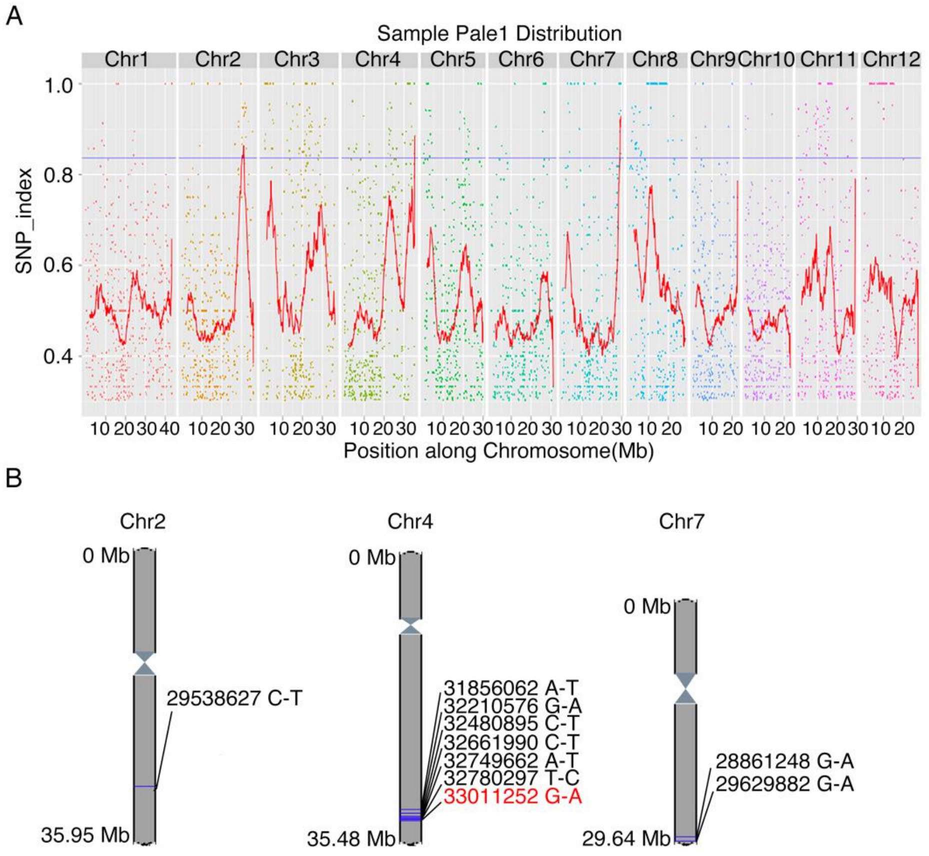

Figure S2. Cloning of *OsEME1*.

(A) BSA-seq analysis of the *k48* group (Pale1). (B) Locations of the candidate genes on chromosomes; the chromosome diagram was downloaded from Ensembl Plants.

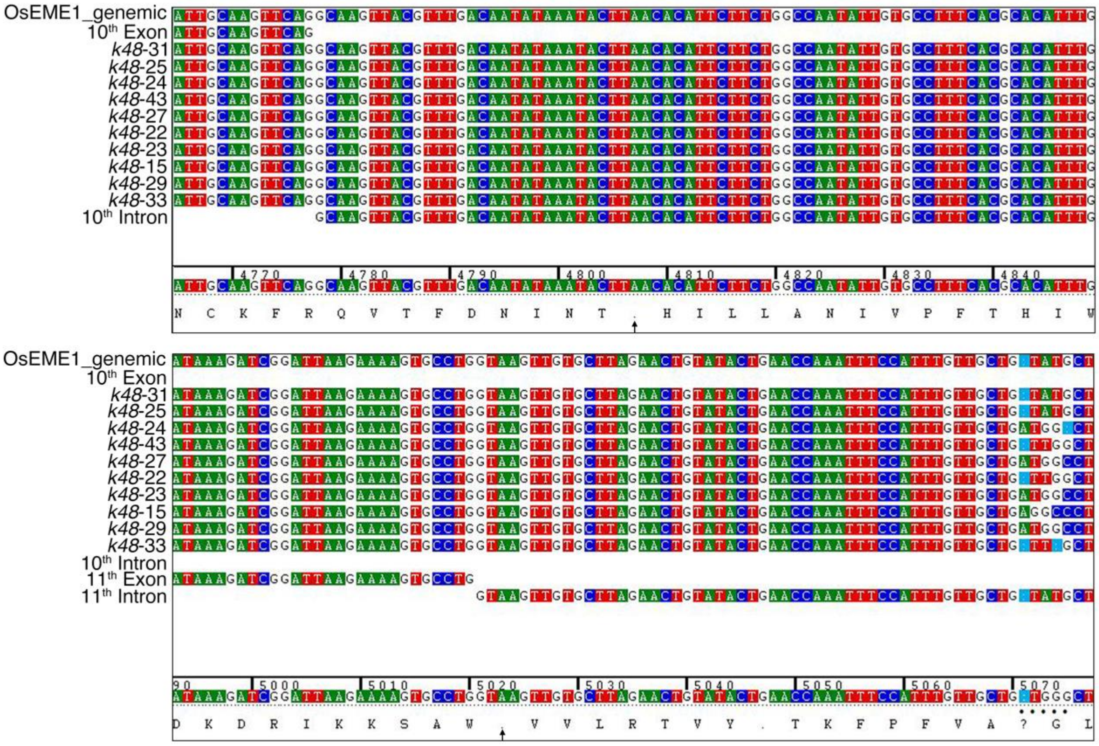

Figure S3. Intron retention and premature translation termination of *OsEME1* in *k48*.

The RNA of *k48* was extracted and reverse-transcribed into cDNA. Primers were specifically designed to amplify the genomic fragment spanning 10th exon to 11th intron. The amplified products were subsequently cloned into the pEASY-Blunt Simple vector. Ten monoclonal colonies were selected for Sanger sequencing, followed by sequence alignment analysis using Sequencher software. The results revealed intron retention events involving both 10th intron and 11th intron in *k48*, which consequently induced premature termination of OsEME1 protein translation. Black arrows indicate the premature termination codon position.

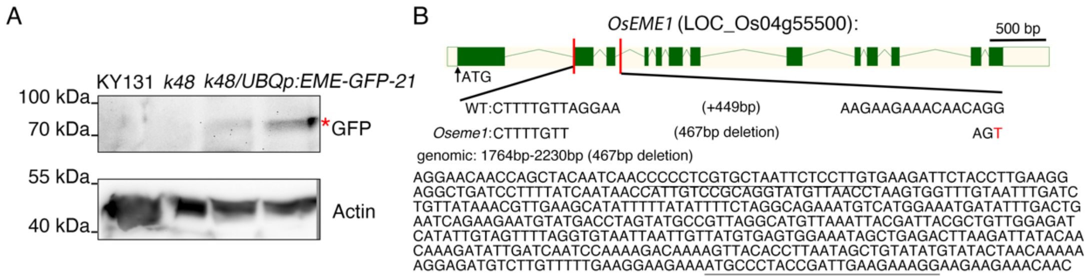

Figure S4. Generation of *OsEME1* CRISPR/Cas9 lines.

(A) Immunoblot analysis of the *k48/UBQp:OsEME1-GFP* complementation lines using anti-GFP antibody. Red asterisk indicates OsEME1-GFP band. Immunoblotting with anti-Actin served as the loading control. (B) Diagram of the *OsEME1* gene structure in wild-type KY131 (WT), and sequence information for the CRISPR/Cas9 mutant *Oseme1* (mut1). Green bars indicate exons, while lines denote introns.

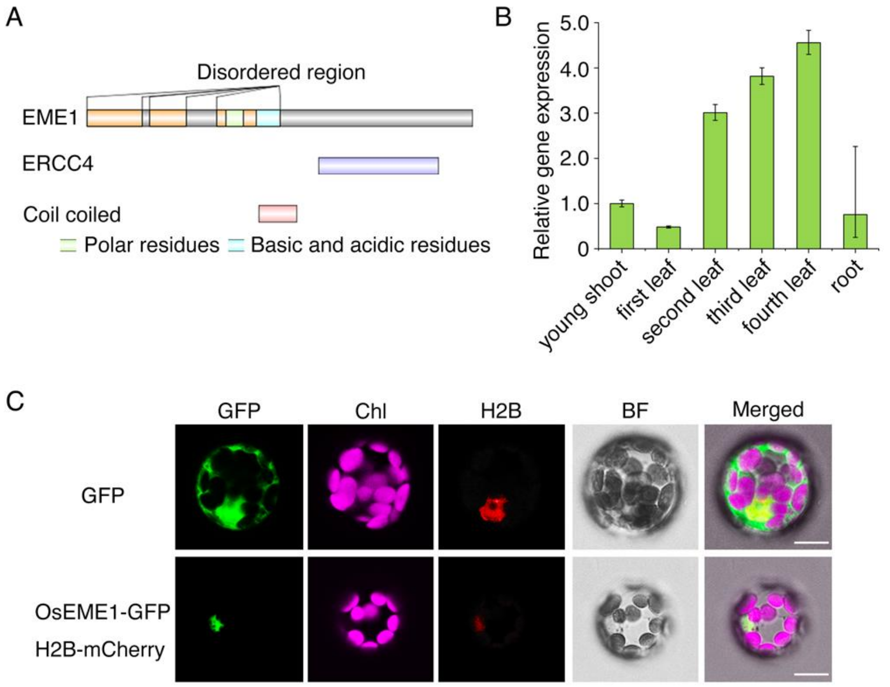

Figure S5. Sequence and expression analysis of *OsEME1*.

(A) Diagram of the regions in EME1 drawn using the IBS (Illustrator for Biological Sequences) website (http://ibs.biocuckoo.org/online.php). (B) Relative *OsEME1* expression in different tissues of plants at the four-leaf stage normalized to the *OsUBQ5* control. Data are mean ± s.d. from three biological replicates. (C) Subcellular

localization of OsEME1-GFP. Co-transformation of H2B-mCherry showing nuclear localization. BF, bright field; Chl, chlorophyll autofluorescence; H2B, fluorescence of H2B-mCherry. Bars, 10 μm.

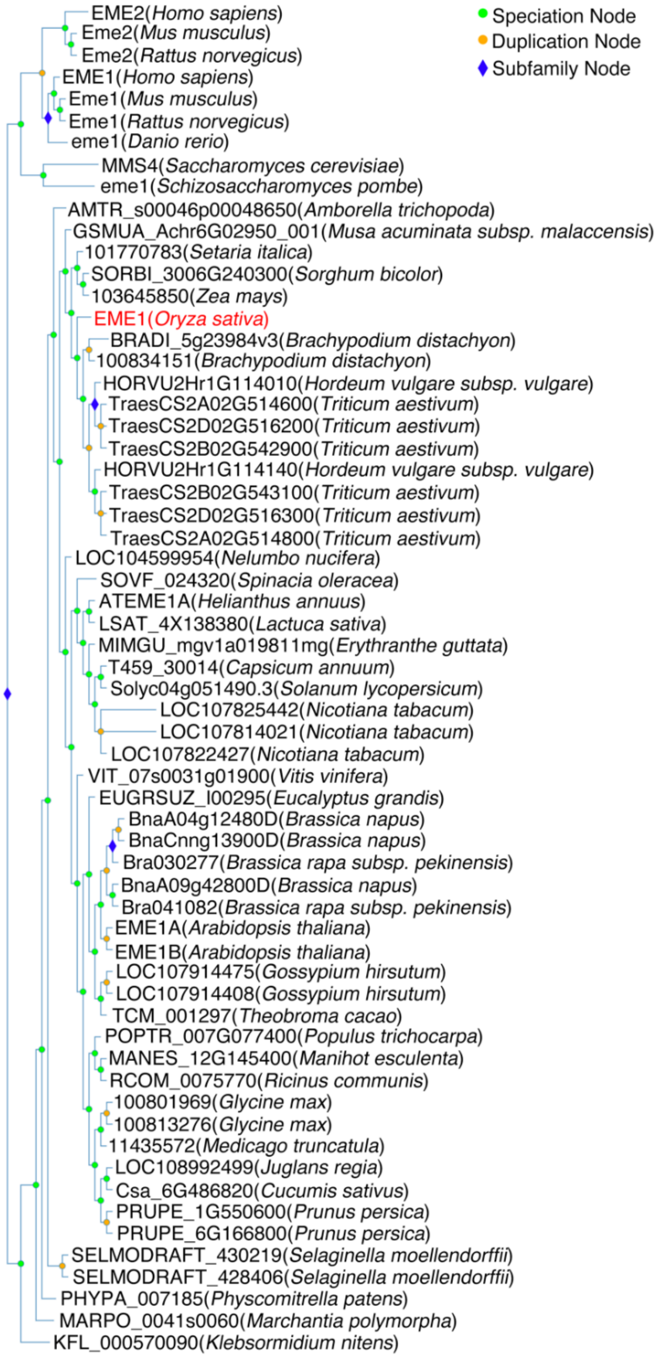

Figure S6. Phylogenetic tree of EME1.

The phylogenetic tree was drawn with PhyloGenes (http://www.phylogenes.org/tree/) using the sequences of 62 genes from 42 organisms.

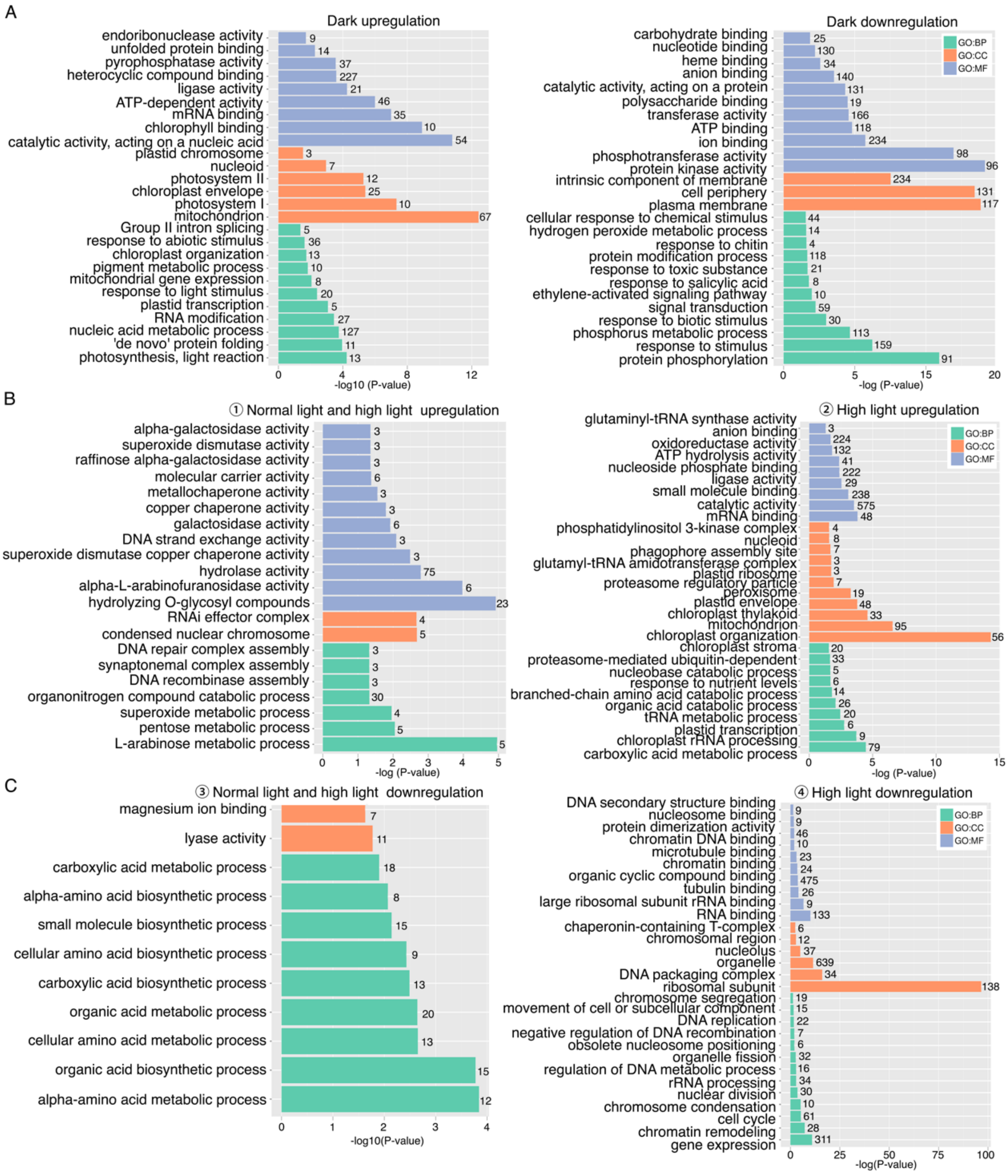

Figure S7. Gene Ontology (GO) analysis of differentially expressed genes (DEGs) in *k48* grown in the dark or light.

(A) GO analysis of upregulated or downregulated genes in *k48* compared to KY131 grown in the dark. (B) GO analysis of upregulated genes in *k48* compared to KY131 grown under normal-light and high-light conditions or only high-light conditions. (C) GO analysis of downregulated genes in *k48* compared to KY131 under normal-light and high-light conditions or only high-light conditions.

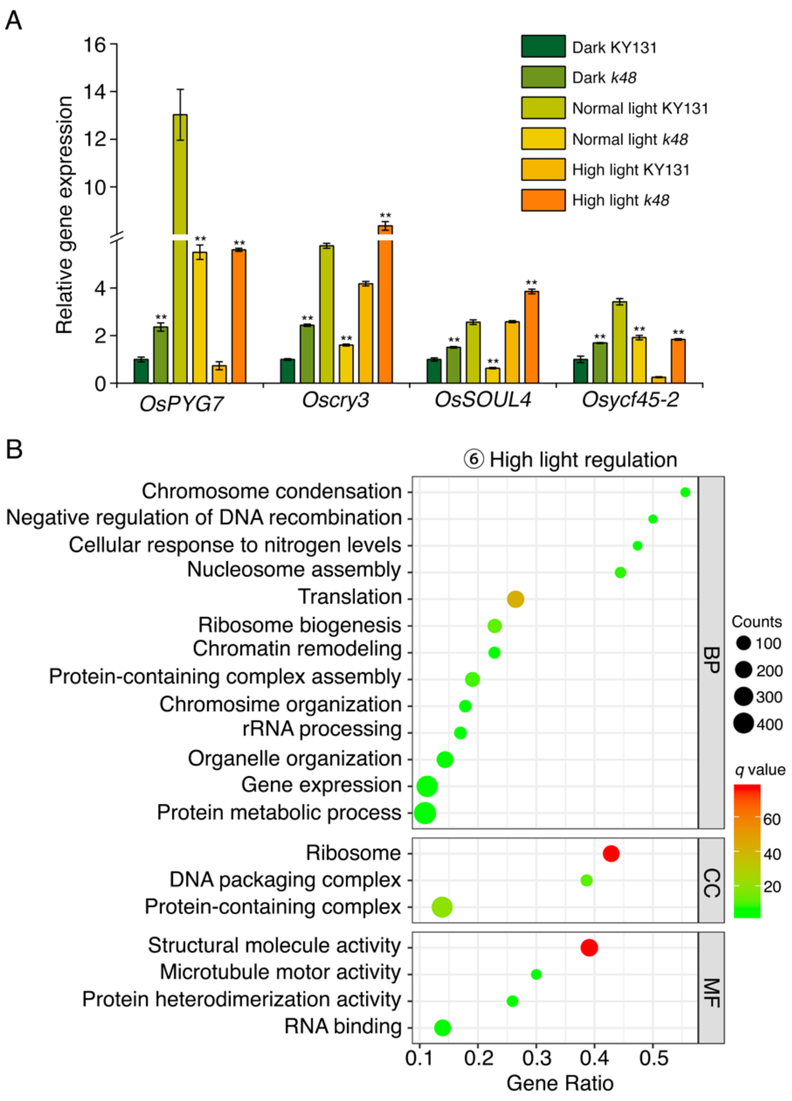

Figure S8. Gene Ontology (GO) and gene expression analyses.

(A) Relative transcript levels of four representative genes in KY131 wild-type and *k48* plants grown under normal-light (NL) conditions or high-light (HL) conditions, as shown in Figure 4. Data are mean ± s.d. from three biological replicates. Asterisks indicate significant differences using Student's *t*-test (**$P < 0.01$). (B) GO analysis of upregulated or downregulated genes in *k48* compared to KY131 under high-light conditions.

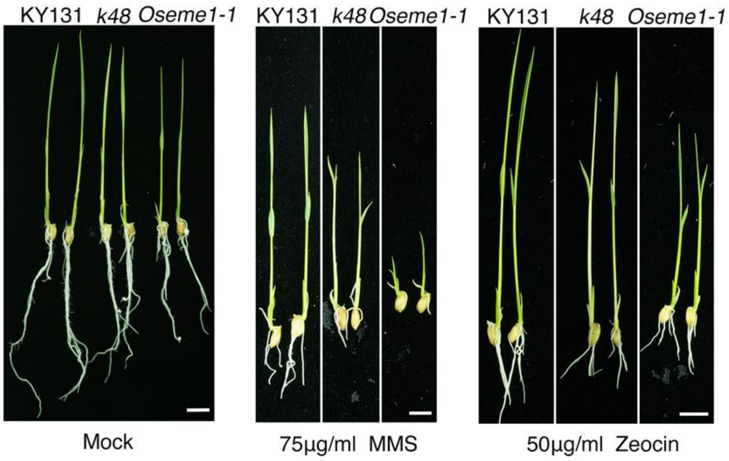

Figure S9. Loss of *OsEME1* increases sensitivity to DNA damage.

Phenotype of seedlings grown in Hoagland's medium without (Mock) or with DNA-damaging agents, 75 μg/mL methyl methanesulfonate (MMS) or 50 μg/mL Zeocin, for 5 days. Bars, 1 cm.

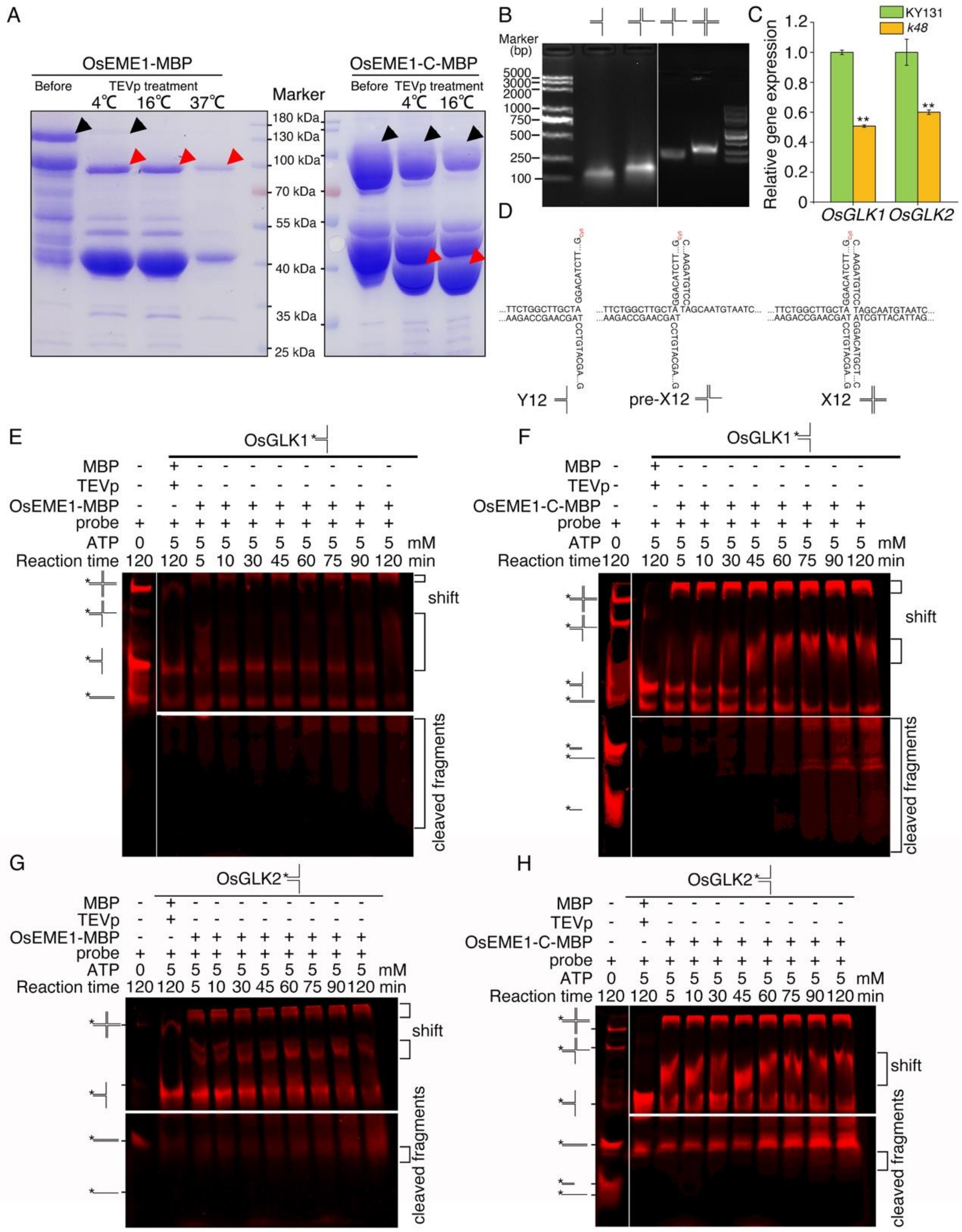

Figure S10. OsEME1 directly binds to and cleaves *OsGLKs* Y12 substrates.

(A) OsEME1 and OsEME1-C recombinant proteins were purified, and the maltose binding protein (MBP) tag was cleaved before the endonuclease activity assay. Black arrows indicate purified OsEME1 or OsEME1-C proteins; red arrows indicate OsEME1 or OsEME1-C without the MBP tag. (B) DNA substrates analyzed by 1.5% agarose gel electrophoresis. (C) Expression analysis of *OsGLK1* and *OsGLK2* in wild-type KY131

and *k48* plants grown under normal light conditions, as shown in Figure 1. Data are mean ± s.d. from three biological replicates. Asterisks indicate significant differences using Student's *t*-test (**P < 0.01). (D) Diagram of the DNA damage substrates including "Y12", "pre-X", "X" substrates. (E and F) The Y12 substrates of *OsGLK1* cleaved by OsEME1 (E) or OsEME1-C (F). (G and H) The Y12 substrates of *OsGLK2* cleaved by OsEME1 (G) or OsEME1-C (H).

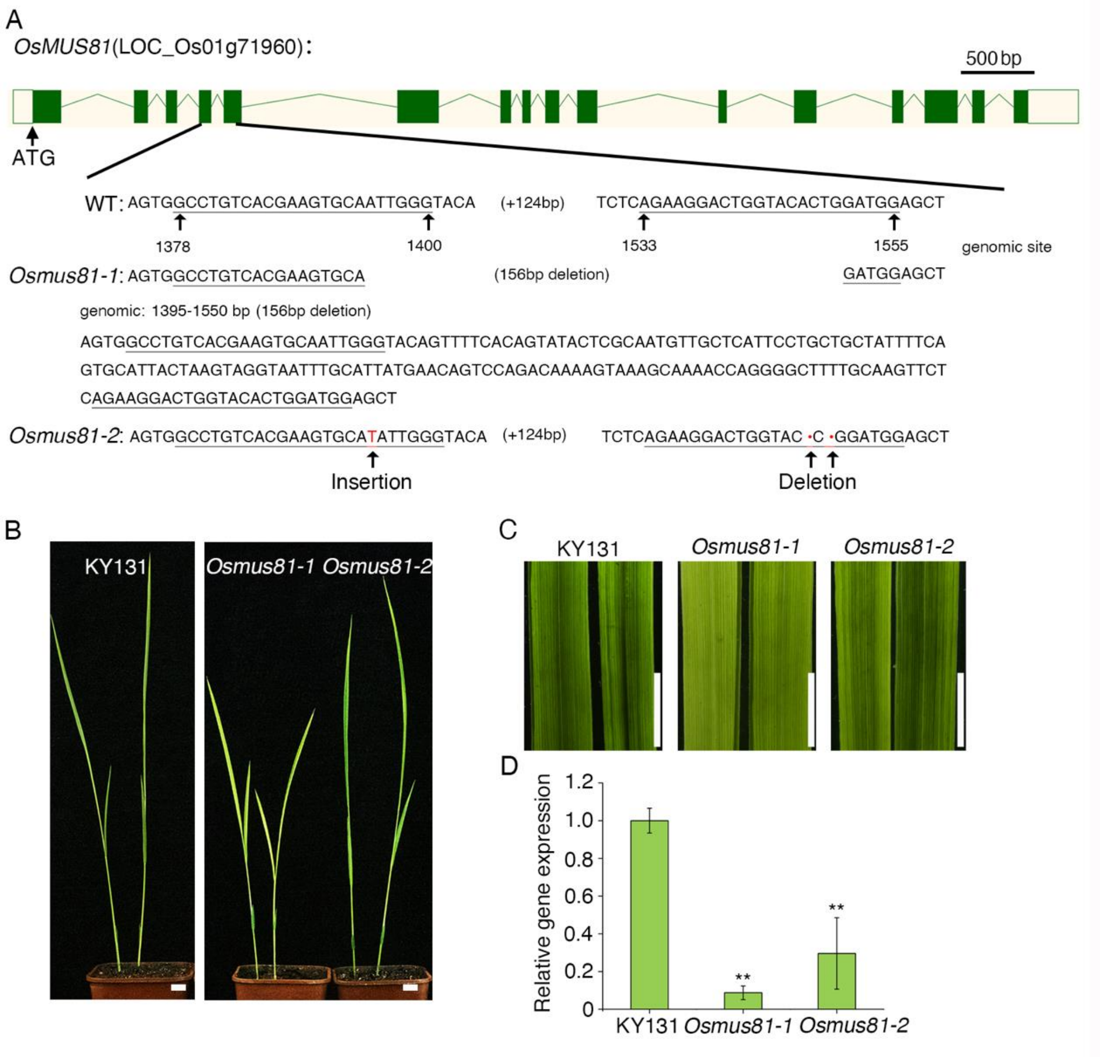

Figure S11. Generation of *Osmus81* CRISPR/Cas9 lines.

(A) Diagram of the *OsMUS81* gene structures in KY131 wild type (WT) and the *Osmus81* CRISPR/Cas9 lines. Green bars indicate exons, while lines denote introns. Sequence information is shown for the two *Osmus81* mutant lines. (B and C) Phenotypes of seedlings (Bar, 1 cm) (B) and leaves (Bar, 0.5 cm) (C) of plants grown under normal light conditions for 12 d. (D) *OsMUS81* expression in the wild-type and mutant lines. Data are mean ± s.d. from three biological replicates. Asterisks indicate significant differences using Student's *t*-test (***P* < 0.01).

Table S1. List of primers used in this study.

	Forward sequence (5'-3')	Reverse sequence (5'-3')
EME1 point mutant	AGCTATTGCTATTGAGAAGAAGT	CAATAGCAATAGCTTGTCCTG
EME1(FL)	GGTACCCAATTGATGGCGTCCCACGC CGC	GGATCCCTCGAGAGCTCCACG CCTATCAACTTCCAC
EME1-D2	GGTACCCAATTGATGGCGTCCCACGC CGCC	GTCGACCTTCAATCGGTAGGG CATTTTCT
EME1-D3	GGTACCCAATTGGCCCTACCGATTGA AGAAAGGA	GGATCCCTCGAGAGCTCCACG CCTATCAACTTCCAC
EME1-D4	GGTACCCAATTGATGCATGAGAAGG GCCTTTGTTT	GGATCCCTCGAGAGCTCCACG CCTATCAACTTCCAC
EME1-D5	AAAATGCCCTACCGATGTATTGTTGC TGAGATTGACTC	TTTTGTAGCTAACTTCGGTAG GGCATTTTCTTCC
EME1-D6	GGTACCCAATTGATGCCCCTTCCCCT CCAGACCCG	GGATCCCTCGAGAGCTCCACG CCTATCAACTTCCAC
EME1 L439V	TGGTACATTAGTGGATCATG	CACTAATGTACCACCACTTA
EME1 P487A	AAGCGTCCAGCAGTGGAAGA	TGCTGGACGCTTCCAACTAT
EME1 E490A	CAGTGGAAGCGGCCTTATGC	CCGCTTCCACTGGTGGACGC
EME1 K494E	GGCCTTATGCGAATTAGCGA	TTCGCATAAGGCCTCTTCCA
EME1 ter	GGTACCCAATTGATGGCGTCCCACGC CGC	GGATCCCTCGAGCTAAGCTCC ACGCCTATCAACTTCCAC
MUS81	CCCGGGGGTACCATGGCGCCGGAGG CGAGG	CTCGAGGTCGACTCCTTCAGC CCAGACAAGCTTGAA
MUS81-D3	GGTACCATAACTGAAGAAGGGAAAT CGACT	CTCGAGGTCGACTCCTTCAGC CCAGACAAGCTTGAA
MUS81-D5	GGTACCGAATTCATGCTTGATACATC AGAAAGGATCAAAACT	CTCGAGGTCGACTCCTTCAGC CCAGACAAGCTTGAA
MUS81 ter	CCCGGGGGTACCATGGCGCCGGAGG CGAGG	CTCGAGGTCGACTTATCCTTC AGCCCAGACAAGCTTGAA
MUS81-FL Y2H	ATGGCCATGGAGGCCAGTGAATTCAT GGCGCCGGAGGCGAG	TCTACGATTCATCTGCAGCTC GAGTCCTTCAGCCCAGACAA G
MUS81-D2 Y2H	ATGGCCATGGAGGCCAGTGAATTCAT GGCGCCGGAGGCGAG	TCTACGATTCATCTGCAGCTC GAGAAGAGCAGTCGATTTCCC TT

MUS81-D3 Y2H	ATGGCCATGGAGGCCAGTGAATTCATAACTGAAGAAGGGAAATCGAC	TCTACGATTCATCTGCAGCTCGAGTCCTTCAGCCCAGACAAG
MUS81-D4 Y2H	ATGGCCATGGAGGCCAGTGAATTCATGATAACTGAAGAAGGGAAATCG	TCTACGATTCATCTGCAGCTCGAGCCTTTCTGATGTATCAAGAGG
MUS81-D5 Y2H	GGTACCGAATTCATGCTTGATACATCAGAAAGGATCAAAACT	TCTACGATTCATCTGCAGCTCGAGTCCTTCAGCCCAGACAAG
EME1-BiFc	TAGAGATCTCGAGCTCAAGCTTCGATGGCGTCCCACGCCGC	CAGGATCCCGGGCCCGCGGTACCtAGCTCCACGCCTATCAACTTCCAC
MUS81-BiFc	TAGAGATCTCGAGCTCAAGCTTCGATGGCGCCGGAGGCGAGG	AGGATCCCGGGCCCGCGGTACCtTCCTTCAGCCCAGACAAGCTTGAA
EME1-D2 LCI	GGTACCCAATTGATGGCGTCCCACGCCGCC	GTCGACCTATTCAATCGGTAGGGCATTTTCT
EME1-D3 LCI	GGTACCCAATTGGCCCTACCGATTGAAGAAAGGA	GGATCCCTCGAGCTAAGCTCCACGCCTATCAACTTCCAC
EME1-D4 LCI	GGTACCCAATTGATGCATGAGAAGGGCCTTTGTTT	GGATCCCTCGAGCTAAGCTCCACGCCTATCAACTTCCAC
EME1-D6 LCI	GGTACCCAATTGATGCCCCTTCCCCTCCAGACCCG	GGATCCCTCGAGCTAAGCTCCACGCCTATCAACTTCCAC
eme1-cas9 test	TGTCCAATATCTCTGGATAGTG	CTCCAGCTTCTTCAATTCT
mus81-cas9 test	GGTATGCTTTGCTTGGGGA	TCAAGAATGTGGCACTGCC
k48-mutant test	GCTCAAGGCACTGCACAGAT	AGGCAAAGCATACAGCAACAAA
EME1-TEV	CAATTGGAGAACCTGTACTTCCAATCCATGGCGTCCCACGCCGC	GGATCCCTCGAGAGCTCCACGCCTATCAACTTCCAC
EME1-D3 TEV	CATATGGATATCGGTACCGAGAACCTGTACTTCCAATCCGCCCTACCGATTGAAGAAAG	GAATTGGTCGACCTCGAGAGCTCCACGCCTATCAACTTCCAC
MUS81-TEV	GGTACCGAGAACCTGTACTTCCAATCCATGGCGCCGGAGGCGAGG	CTCGAGGTCGACTCCTTCAGCCCAGACAAGCTTGAA
DNA substrate X1	GACGCTGCCGAATTCTGGCTTGCTAGGACATCTTTGCCCACGTTGACCCG$_{Cy5}$	
DNA substrate X2	CGGGTCAACGTGGGCAAAGATGTCCTAGCAATGTAATCGTCTATGACGTC	

DNA substrate X3	GACGTCATAGACGATTACATTGCTA GGACATGCTGTCTAGAGACTATCGC	
DNA substrate X4	GCGATAGTCTCTAGACAGCATGTCC TAGCAAGCCAGAATTCGGCAGCGTC	
GLK1 X1	Cy5CGTCGATGACATAGACTTCGGGG ACTTCTTCCTGAGGCTGGAGGACGG TG	
GLK1 X2	GAAGTCGGTGAAGATCTCGGCCGGG GTCCCCGAAGTCTATGTCATCGACG	
GLK1 X3	TCGACCTCGAGGTCCGGGAGCACAT CACCGTCCTCCAGCCTCAGGAAGAA	
GLK1 X4	ATGTGCTCCCGGACCTCGAGGTCGA CCCGGCCGAGATCTTCACCGACTTC	
GLK2 X1	Cy5GAAGAAGAAGGACGACGAGGAA AGGTCGTCGTCGTTGCCGGAGGAGA AAG	
GLK2 X2	CTCCGTCGTCACCGCGCTCAGGACC CCTTTCCTCGTCGTCCTTCTTCTTC	
GLK2 X3	TCGTCGCCGCCGCCGTTCTTCGCGTC TTTCTCCTCCGGCAACGACGACGA	
GLK2 X4	ACGCGAAGAACGGCGGCGGCGACG AGGTCCTGAGCGCGGTGACGACGGA G	
EME1 RT	TGCTATTCCCGGAGTCAGTC	TTTAGAGCATGCTGGTCCGA
MUS81 RT	ACTTCTCAAGTTTGCCTGACC	GCAAGTGCAGCTTCTTCTGT
EME1 cas9 site RT	ACCATTGTCCGCAGGTATGT	GCCTAACGGCATACTAGGTC A
MUS81 cas9 site RT	TATTGAGCTCACGCGTTTGG	GGATCAGCGTCAGTGCAATC
psaA	CCTAAGGAGATACCACTTCCTCATG	TTGCTCGTTCGGCAAAACTA
psaB	GGTCAACCCGCTGTGGAA	AAACCCCAGAATAGGCGATA TTC
psaC	GGATGGATGTAAAGCCAAGCA	GCGGATTCGCATCTCTTACAA
psbA	CGTGAGCCTGTTTCTGGTTCT	GCAGCTTCCCAAATTGGGTAA
psbB	TGCCGGAACTATGTGGTATGG	CCCTGATCCCACTGATAACGA
psbC	CATACAACCTTGGCAAGAACGA	CGCCACCCACAGAATTTAAA G
rbcL	GCGATCTTGCTCGTGAAGGTA	TTCACAAGCTGCGGCTAGTTC
rpl12	CGAAACCTAGAAATCGATCACTGAT	TGCCGTTACTCAACAGTTTTC

		TG
rpl14	AGTGCCCCAAATGCCTCTAGA	ACCGCTGCATTGTCGTCATAG
rpl33	GCAATGTGTGAGTTGTGTTCGA	TCGGCATGAATCGTATGCTTAC
rpl36	TGTGTGAGTTGTGTTCGAAAAGG	GTATTGTGGCGATTCTTTTGAGTACT
rps8	TTATACCTCGAGAAGTAGAAAGAATAGCAA	CGAATTTATGCCAACTATCAAGGA
ndhD	TTAATTAATCAATCATACCCTGCAACA	TGGGTATCTGGTAACCACGTATGT
ndhE	AGCCGCCAATTAAAAGGAGA	CGATGGATGGAAGAAAGAATGG
ndhF	CCCTCTTCTCCCACTTCCAGTTA	GACAGGTGAACTGAGAATACCATAGC
PYG7	TCGTCATCCGTCAGGTTCTT	GCACTTGCATCACCACTTCT
cry3	GCGACAGGTTTCATGTCCAA	TTGCCAACTCCTGCTCCATA
SOUL4	GTGAAGGAGAAGGCGGAGT	AGAGTAAACGGCGGGTTGTA
ycf45-2	ACAGCTCACGGAGAACATCT	CAGTAGTGTCGCTCCCTCAT
Actin RT	CCTTCAACACCCCTGCTATG	CAATGCCAGGGAACATAGTG
UBQ5 RT	ACCACTTCGACCGCCACTACT	ACGCCTAAGCCTGCTGGTT
Hyg	AGTACTTCTACACAGCCATC	CTCTCTCGAGCTTTCGCAGATC
Cas9	AACAGGCTTTCTGACTACGACG	GGAGCTCCTTGACGGACTTGAG
